# Integrated bioinformatics and machine learning reveal pan-apoptosis and immune infiltration signatures in diabetic nephropathy

**DOI:** 10.3389/fimmu.2025.1659065

**Published:** 2025-12-17

**Authors:** Yu Liu, Wenqian Lu, Zhicong Xiang, Yuli Shen, Baoyi Ni, Hequn Zou, Xiaofei Shao

**Affiliations:** 1 Department of Nephrology, The Chinese University of Hong Kong, Shenzhen Medical Centre, Shenzhen, China; 2 Guangdong Key Laboratory for Biomedical Measurements and Ultrasound, Imaging School of Biomedical Engineering, Shenzhen University Health Science Center, Shenzhen, China; 3 The Chinese University of Hong Kong-Shenzhen, Shenzhen, China; 4 Department of Nephrology, Shenzhen Luohu People's Hospital, Shenzhen, China; 5 Nephrology Department of The Second Affiliated Hospital, School of Medicine, The Chinese University of Hong Kong, Shenzhen, China; 6 Department of Oncology, Heilongjiang University of Chinese Medicine, Harbin, China; 7 Department of Nephrology, The Third Affiliated Hospital, Southern Medical University, Guangzhou, China

**Keywords:** diabetic nephropathy, PANoptosis, bioinformatics, machine learning, immune infiltration

## Abstract

**Background:**

Diabetic nephropathy (DN) is one of the vascular complications of diabetes and a leading cause of end-stage renal disease (ESRD) and mortality in diabetic patients. PANoptosis has been defined a unique form of programmed cell death that integrates pyroptosis, apoptosis, and necroptosis. However, the role of other biomarkers in modulating PANoptosis and their impact on DN remains unexplored.

**Objective:**

This study aimed to explore panoptosis-related genes and potential therapeutic drugs in DN.

**Methods:**

We downloaded DN datasets from the GEO database and identified differentially expressed genes (DEGs) through integrated differential expression analysis and weighted gene co-expression network analysis (WGCNA). The intersection between DN-related DEGs and panoptosis-related genes was obtained, and LASSO and SVM machine learning algorithms were applied to screen candidate biomarkers. The area under the receiver operating characteristic curve (AUC) was calculated for evaluation. Validation was performed using the merged dataset of GSE30529 and GSE4713. The CIBERSORT algorithm was used to assess immune cell infiltration, and Spearman correlation analysis was conducted to examine the association of biomarker genes. The Kidney Integrative Transcriptomics database was employed to explore the distribution of core genes across 12 cell populations. Potential drug molecules interacting with core genes were screened using the DSigDB database on the Enrichr platform, and molecular docking was performed using AutoDock Vina to evaluate binding affinity. The qRT-PCR was used to validate the expression of these hub mitochondria-related genes.

**Results:**

Analysis of the DN dataset yielded 17 intersecting genes. Gene Ontology (GO) and Kyoto Encyclopedia of Genes and Genomes (KEGG) enrichment analyses revealed that these genes were significantly associated with immune and inflammatory responses, pyroptosis, extrinsic apoptosis, necroptosis, and related pathways. Using LASSO and SVM machine learning algorithms, eight candidate biomarkers were identified: CD44, CRIP1, CEBPB, TNFRSF1B, CAV1, IGF1, GZMB, and LY96. ROC curve analysis demonstrated that these biomarkers had strong diagnostic value for DN patients. Further investigation into immune infiltration in DN samples using CIBERSORT showed that core genes were closely related to dendritic cells (resting), macrophages (M1), mast cells (activated), neutrophils, T cells (CD4 memory activated, CD4 memory resting, CD8, and gamma delta). Drug screening via DSigDB on Enrichr identified imatinib as a significantly enriched drug interacting with core genes, and molecular docking confirmed its strong binding affinity.

**Conclusion:**

Through comprehensive bioinformatics approaches, this study identified CD44, CRIP1, CEBPB, TNFRSF1B, CAV1, IGF1, GZMB and LY96 as potential diagnostic biomarkers for DN, providing new insights into disease diagnosis.

## 1 Introduction

Diabetic nephropathy (DN) is one of the vascular complications of diabetes and a leading cause of end-stage renal disease (ESRD) and mortality in diabetic patients (1). In developed countries, approximately 50% of ESRD cases are attributed to DN. In China, DN has surpassed primary glomerular diseases to become the primary etiology of chronic kidney disease (CKD), imposing a heavy burden on patients, families, and society (2, 3). Currently, the primary treatment strategies for DN include strict glycemic control, the use of angiotensin-converting enzyme inhibitors (ACEIs) or angiotensin II receptor blockers (ARBs) to manage blood pressure, and interventions to mitigate glomerulosclerosis and interstitial fibrosis. However, 20%–30% of patients still experience progressive renal function decline, ultimately progressing to ESRD (4).

The pathogenesis of DN is complex, involving genetic factors, hyperglycemia, hemodynamic abnormalities, pyroptosis, autophagy, and other mechanisms. Elevated blood glucose levels induce metabolic disturbances and renal hemodynamic changes, leading to disordered glucose metabolism in renal tissues, increased glomerular capillary permeability, extracellular matrix accumulation, and progressive glomerulosclerosis (5, 6). Under hyperglycemic conditions, angiotensin II (Ang II) production increases, causing systemic arteriolar constriction, lipid peroxidation, and subsequent accumulation in renal cells. Overactivation of the renin-angiotensin-aldosterone system (RAAS) and hyperfiltration states promote apoptosis, pyroptosis, and dysregulated autophagy in renal cells, triggering fibroblast transdifferentiation, glomerulosclerosis, and interstitial fibrosis (7). Autophagy, a lysosome-dependent self-protective mechanism for degrading damaged proteins and macromolecules, is suppressed in DN, impairing the clearance of metabolic waste and leading to toxic substance accumulation in renal tissues (8, 9). Studies have shown that GSDMD-mediated pyroptosis plays a critical role in diabetes-induced kidney injury. In DN patients, the GSDMD-dependent pyroptosis pathway is activated by the TLR4/NF-κB signaling cascade, resulting in renal tubular epithelial cell damage (10).

The role of inflammation in DN has been well established. The immune microenvironment of DN is characterized by chronic inflammation induced by hyperglycemia, including the accumulation of pro-inflammatory macrophages, activation of inflammatory pathways, and increase in cytokines, all of which lead to renal damage and fibrosis in DN (11). Furthermore, this cascade reaction also prepares the environment for programmed cell necrosis (PANoptosis), a unique form of programmed cell death that integrates pyroptosis, apoptosis, and necroptosis. Pyroptosis is a highly inflammatory, gasdermin-mediated lysis process triggered by inflammasome activation; apoptosis is a caspase-dependent non-lytic mechanism that enables controlled cell removal; necroptosis is a regulated form of necrotic death driven by RIPK1, RIPK3, and MLKL. PANoptosis represents a comprehensive cell death program that combines the characteristics of all three pathways through the assembly of the multi-protein PANoptosome complex, enabling coordinated activation under immune or metabolic stress conditions (12). This process is triggered by the activation of inflammasome molecules, such as NLR family pyrin domain-containing 3 (NLRP3), which is absent in melanoma 2 and involved in the innate immune response to microbial infections and changes in cellular homeostasis (13). Studies have shown that high glucose levels can activate the NLRP3 inflammasome, leading to kidney damage in metabolic-related kidney diseases (14). On the other hand, pyroptosis, apoptosis, and necroptosis can lead to renal cell loss, fibrosis development, and stimulation of renal inflammatory responses (15). Traditionally, these pathways were considered independent; however, the discovery of PANoptosis reveals their interconnected regulation via the PANoptosome, acting as a molecular hub. This suggests that these cell death pathways are not mutually exclusive but can be co-regulated, unveiling a new dimension in cell death mechanisms. Recent studies indicate that TRAIL induces podocyte PANoptosis via the DR5 receptor, and genetic ablation of TRAIL mitigates podocyte injury and DN progression (16). However, the role of other biomarkers in modulating PANoptosis and their impact on DN remains unexplored.

**Figure 1:**
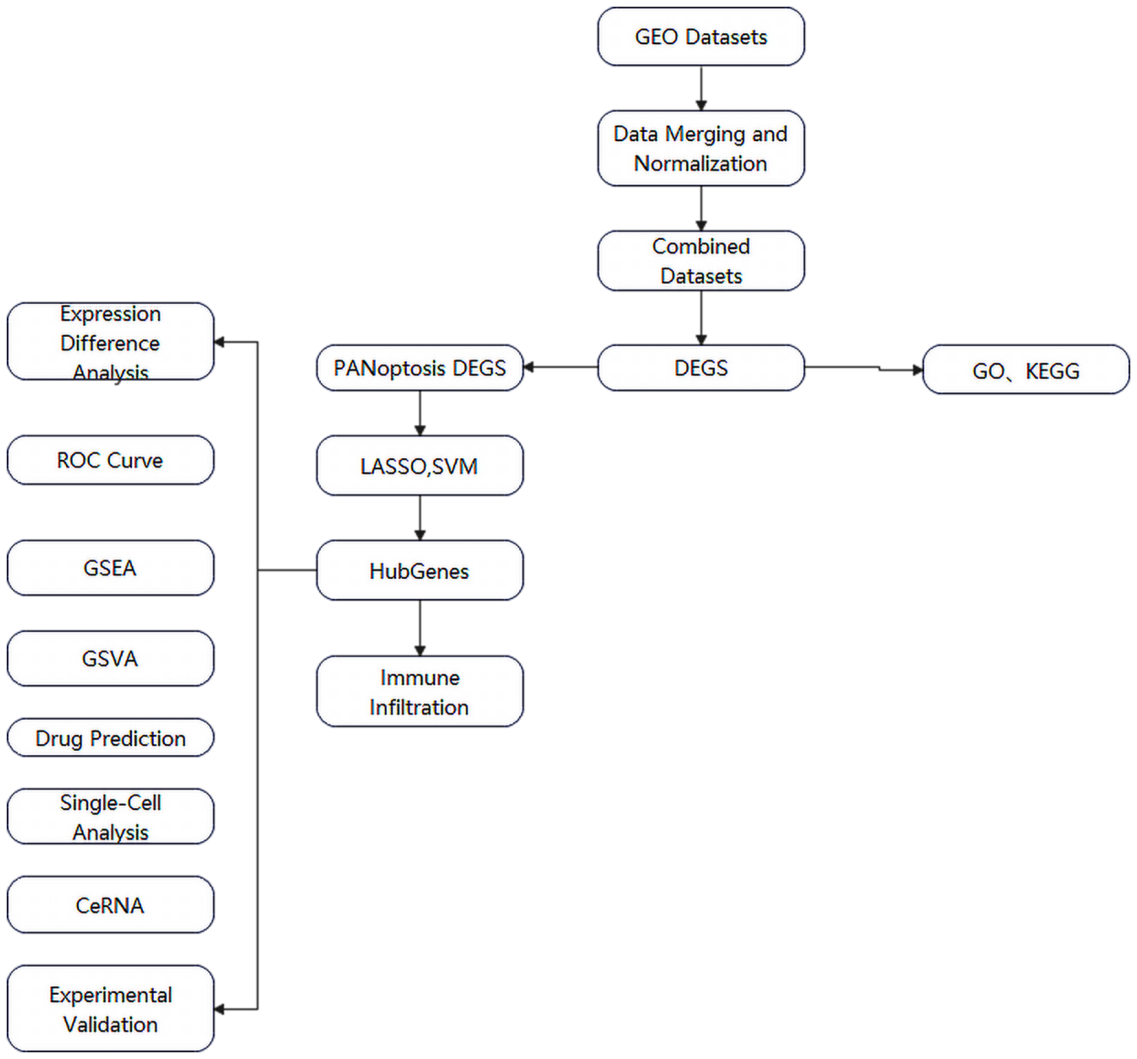
Flow chart.

Therefore, this study aims to identify PANoptosis-related genes in DN renal tissues using bioinformatics approaches, apply machine learning to screen key biomarkers, and evaluate immune cell infiltration patterns via the CIBERSORT algorithm. By integrating these strategies, we seek to elucidate the molecular mechanisms underlying PANoptosis in DN, identify potential diagnostic biomarkers, and provide a theoretical foundation for early detection and targeted therapy. The workflow of this study is illustrated in **Figure 1**.

## 2 Methods

### 2.1 Data acquisition and preprocessing

The Gene Expression Omnibus (GEO) database (https://www.ncbi.nlm.nih.gov/geo/), established by the National Center for Biotechnology Information (NCBI) in 2000, is a public repository containing gene expression data including microarray and high-throughput sequencing data submitted by research institutions worldwide. In this study, we applied the following inclusion criteria: (1) human subjects; (2) inclusion of both patient and healthy control groups; (3) sample size ≥10 for both disease and control groups; (4) kidney tissue or blood samples. Based on these criteria, we downloaded mRNA expression profiles (GSE47184 (17), GSE104948 (18), GSE30529 (19), GSE47183 (20)) and divided them into a training set (GSE47184, GSE104948) and a validation set (GSE30529, GSE47183). In the identified dataset, we selected the balanced dataset as the training group, as it avoids bias toward overfitting errors of the majority class, accurately evaluates the performance of machine learning models, enhances the model’s generalization ability, and ensures reliable predictions for the test dataset. The remaining dataset was designated as the test group. Subsequently, we merged all datasets based on common genes in the training group. Then, we randomly divided the samples into a training set and a validation set at a ratio of 7:3. Afterwards, we corrected batch effects in the training and validation sets using the “ComBat” algorithm18 from the “sva” R package19 (version 3.46.0). The training set helps identify potential diagnostic biomarkers, while the validation set is used to verify the analysis results. In addition, the test group is used to evaluate the diagnostic performance of the biomarkers Following SVA correction, the ComBat algorithm was further applied to enhance inter-sample comparability. Data were then log-transformed to improve distribution and analytical efficiency. Finally, boxplots and principal component analysis (PCA) were used to visualize the batch-corrected expression profiles.

### 2.2 Screening of differentially expressed genes

DEGs refer to gene subsets that show significant expression differences under different experimental conditions, which can be used to analyze biological functions or predict treatment effects. Using the “limma” package with thresholds of |logFC| > 0.585 and adjusted p-value < 0.05 (21), we identified DEGs associated with diabetic nephropathy. The pheatmap and ggplot2 packages were then employed to generate volcano plots and heatmaps for visualizing these DEGs.

### 2.3 Acquisition of PANoptosis-related genes

PANoptosis-related genes include key regulators of apoptosis, pyroptosis, and necroptosis. This gene set was obtained from the literature published by Li Li (22). The genes included IL-17, HIF-1, p53, NF-kappa B, TGF-beta, Toll-like receptor, ITGAM, S100A8, CD14, and SFRP2 (16).

### 2.4 Weighted gene co-expression network analysis

WGCNA aims to identify co-expressed gene modules and explore their associations with phenotypes of interest, as well as to identify hub genes within networks. Using the “WGCNA” R package, we constructed gene co-expression networks from diabetic nephropathy datasets and removed outlier samples through cluster tree analysis. A similarity matrix was generated by calculating correlation coefficients between gene pairs. To ensure scale-free network construction, an appropriate soft threshold was selected to transform the similarity matrix into an adjacency matrix, followed by generation of a topological overlap matrix (TOM) to evaluate the average network connectivity of each gene. Genes with similar expression patterns were grouped into modules using dynamic tree cutting. Module eigengenes (MEs) were used to represent the expression profiles of each module and to assess module-phenotype associations. The module showing the highest absolute correlation coefficient was selected as the key module for further analysis. Module membership (MM) reflects the correlation between gene expression values and the corresponding ME, while gene significance (GS) represents the correlation between gene expression and phenotype traits (represented by different colors). Genes in the lightgreen module were considered unassigned to any module.

### 2.5 Venn diagram analysis

We identified intersecting genes among DEGs, WGCNA-derived genes, and PANoptosis-related genes, and visualized these intersections using jvenn (https://jvenn.toulouse.inrae.fr/app/index.html).

### 2.6 GO and KEGG enrichment analyses

Functional enrichment analyses were performed on the intersecting genes using Gene Ontology (GO) and Kyoto Encyclopedia of Genes and Genomes (KEGG) pathway analyses. GO analysis covered three categories: biological processes (BP), molecular functions (MF), and cellular components (CC). Enrichment results with p-value < 0.05 and adjPvalFilter = 1 were considered statistically significant. The R packages ‘org.Hs.eg.db’ was used for gene ID conversion and ‘clusterProfiler’ for enrichment analysis, with visualization performed using ‘RColorBrewer’, ‘enrichplot’, and ‘ggplot2’.

### 2.7 Machine learning-based identification of core genes

To identify PANoptosis-related core genes in diabetic nephropathy, we employed two machine learning algorithms: least absolute shrinkage and selection operator (LASSO) via the “glmnet” package and support vector machine (SVM) via the “caret” package.

To obtain results closest to ideal, we employed Lasso regression analysis with the assistance of the glmnet tool and cross-validation. Compared to previous methods, this approach is highly effective in minimizing overfitting, thereby enhancing the model’s ability to handle the complexity of biological data. SVM is a machine learning supervised classification algorithm that distinguishes sample types by estimating the probability that a sample belongs to a certain category. For the training set, SVM method is used to construct an SVM classifier (core: Sigmoid Kernel; Cross validation: 100x cross validation. The intersection of genes selected by both methods was considered as consensus core genes, visualized using Venn diagrams.

### 2.8 Evaluation and validation of core gene prediction models

To assess the diagnostic performance of core genes, we performed differential expression analysis using the “limma” package and generated boxplots using “ggpubr” to display expression differences between groups. Receiver operating characteristic (ROC) curves were plotted using the “pROC” function to evaluate classification performance, with the area under the curve (AUC) serving as a key metric (range 0-1, higher values indicating better performance). For external validation, we used the combined GSE30529 and GSE47183 datasets to reassess gene expression levels and diagnostic value through boxplots and ROC curves.

### 2.9 Gene set enrichment analysis

Single-gene GSEA was performed to identify regulatory pathways and biological functions associated with core gene expression. Samples were divided into high- and low-expression groups based on median core gene expression levels. The c2.cp.kegg.v2023.1.Hs.symbols.gmt dataset from the Molecular Signatures Database (MSigDB) was used for pathway analysis (significance threshold p<0.05), with results visualized using the enrichplot package.

### 2.10 Gene set variation analysis

GSVA is a non-parametric, unsupervised method for evaluating changes in gene set enrichment patterns across samples. Using the “GSVA” R package, we performed functional enrichment analysis on diabetic nephropathy samples with “c2.cp.kegg.v7.4.symbols.gmt” from MSigDB as reference. Adjusted p-value < 0.05 was set as the significance threshold for inter-group differences.

### 2.11 Immune infiltration analysis

The “CIBERSORT” R package was used to estimate proportions of 22 immune cell types in each training set sample, examining differences in immune microenvironment between diabetic nephropathy and control groups. Wilcoxon tests (p<0.05) identified differentially abundant immune cell types, visualized using violin plots (vioplot package). Spearman correlation analysis (p<0.05) assessed relationships between immune cells and core genes, with results displayed as heatmaps. CIBERSORT employs Monte Carlo sampling to estimate the p-value, which evaluates the confidence level of the deconvolution results for each sample. In this study, only samples with p-values < 0.05 were included in the final analysis to ensure the robustness and accuracy of the inferred immune cell proportions.

### 2.12 Drug prediction and molecular docking

Gene target-based drug screening represents a novel strategy that can accelerate drug development. Using the DSigDB database (http://dsigdb.tanlab.org/DSigDBv1.0/) via the Enrichr platform, we identified drug molecules interacting with core genes (adjusted p-value < 0.05 considered significant). To validate binding affinities between active compounds and core targets, we performed molecular docking. Key compounds were retrieved from PubChem, with chemical structures drawn using ChemDraw and 3D structures generated and energy-minimized (“Calculation-MM2-Minimize Energy-Run”) in ChemBio3D (saved as SDF files). PyMOL converted compounds to PDB format. Crystal structures of core targets were obtained from the Protein Data Bank (PDB; http://www.rcsb.org/), prepared (water removal, hydrogen/charge addition) using AutoDock Vina, and saved as PDB files. AutoDock Vina performed molecular docking, with the lowest binding energy conformations selected for further analysis. PyMOL 1.8 provided 3D visualization of compound-target interactions.

### 2.13 Single-cell RNA sequencing analysis

Using the Kidney Integrative Transcriptomics (K.I.T.; http://humphreyslab.com/SingleCell/) database, we analyzed single-cell sequencing data of core genes in diabetic nephropathy samples and visualized the results.

### 2.14 Competing endogenous RNA network prediction

ceRNA networks are established through miRNA-mediated interactions, where miRNAs bind to miRNA response elements, forming regulatory relationships between target mRNAs and lncRNAs that play crucial roles in gene expression regulation. We predicted core gene-related miRNAs using miRanda, miRDB and TargetScan databases, and identified corresponding lncRNAs via spongeScan. The ceRNA regulatory network was constructed using Cytoscape 3.9.1.

### 2.15 Animal experiments

#### 2.15.1 Animal model

Six four-week-old C57BL/KsJ-db/db mice and six db/m mice were purchased and housed in the temperature- controlled environment with a 12-h light/dark cycle, free access to food and water. At the age of 20 weeks, mice were sacrificed and blood, urine, and kidney samples were collected. The kidney was quickly frozen in liquid nitrogen and kept at -80 °C for qRT-PCR. The research has obtained ethical approval from the Ethics Committee of Shenzhen University (Ethics Approval No.: KYLL-20240903A).

#### 2.15.2 Biochemical analysis

Blood urea nitrogen (BUN), and serum creatinine (CREA) were determined with an automatic biochemistry analyzing machine (Hitachi 7600, Tokyo, Japan).

#### 2.15.3 Real-time PCR

Total RNA from mouse kidneys was isolated using TrIzol reagent (Invitrogen) and quantified using a Nanodrop spectrophotometer. Complementary DNA (cDNA) was generated using the PrimeScriptTM RT Reagent Kit (Takara, Otsu, Japan) for mRNA analysis. A TB Green Fast qPCR kit (Takara, Otsu, Japan) was used for real‐time quantitative PCR. β-actin served as the internal control. The primers used for PCR are listed as follows (**Table 1**).

**Table 1:** Primers used for real-time PCR.

Gene	Forward	Reverse
LY96	CGCTGCTTTCTCCCATATTGA	CCTCAGTCTTATGCAGGGTTCA
GZMB	CCACTCTCGACCCTACATGG	GGCCCCCAAAGTGACATTTATT
IGF1	GTGAGCCAAAGACACACCCA	ACCTCTGATTTTCCGAGTTGC
CAV1	ATGTCTGGGGGCAAATACGTG	CGCGTCATACACTTGCTTCT
TNFRSF1B	ACACCCTACAAACCGGAACC	AGCCTTCCTGTCATAGTATTCCT
CRIP1	AAGTGCGACAAGGAGGTGTAT	AGAGGTCAGTGTCTTTCCACATT
CD44	CACCATTGCCTCAACTGTGC	TTGTGGGCTCCTGAGTCTGA
CEBPB	GTTTCGGGACTTGATGCAAT	CCCCGCAGGAACATCTTTA
β-actin	GGCTGTATTCCCCTCCATCG	CCAGTTGGTAACAATGCCATGT

### 2.16 Data analysis

All data were analyzed using R software. For continuous variables with non-normal distributions, the Mann-Whitney U test (Wilcoxon rank-sum test) was employed to assess intergroup differences. Potential correlations between variables were determined using Pearson correlation coefficients. A p-value < 0.05 was considered statistically significant.

## 3 Results

### 3.1 Dataset integration

**Figure 2:**
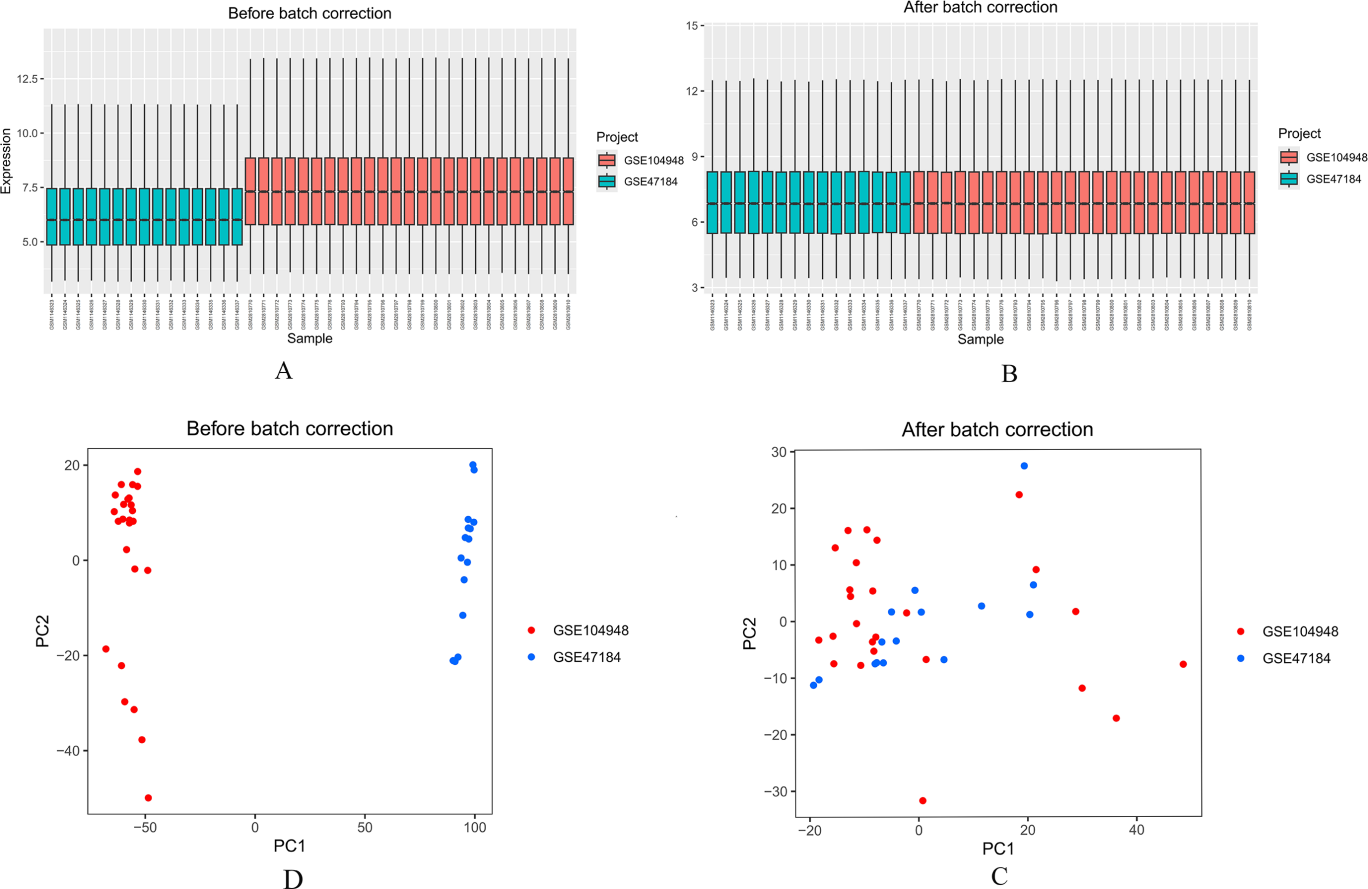
Batch effect removal in diabetic nephropathy datasets. **(A)** Boxplot distribution of merged diabetic nephropathy dataset before normalization. **(B)** Boxplot distribution of merged diabetic nephropathy dataset after normalization. **(C)** PCA plot of merged diabetic nephropathy dataset before normalization. **(D)** PCA plot of merged diabetic nephropathy dataset after normalization.

Boxplots and principal component analysis (PCA) demonstrated substantial elimination of batch effects in the diabetic nephropathy datasets following batch effect correction. The results revealed consistent statistical characteristics post-correction, confirming effective batch effect removal and ensuring the reliability of subsequent analyses (**Figure 2**).

### 3.2 Identification of differentially expressed genes

**Figure 3:**
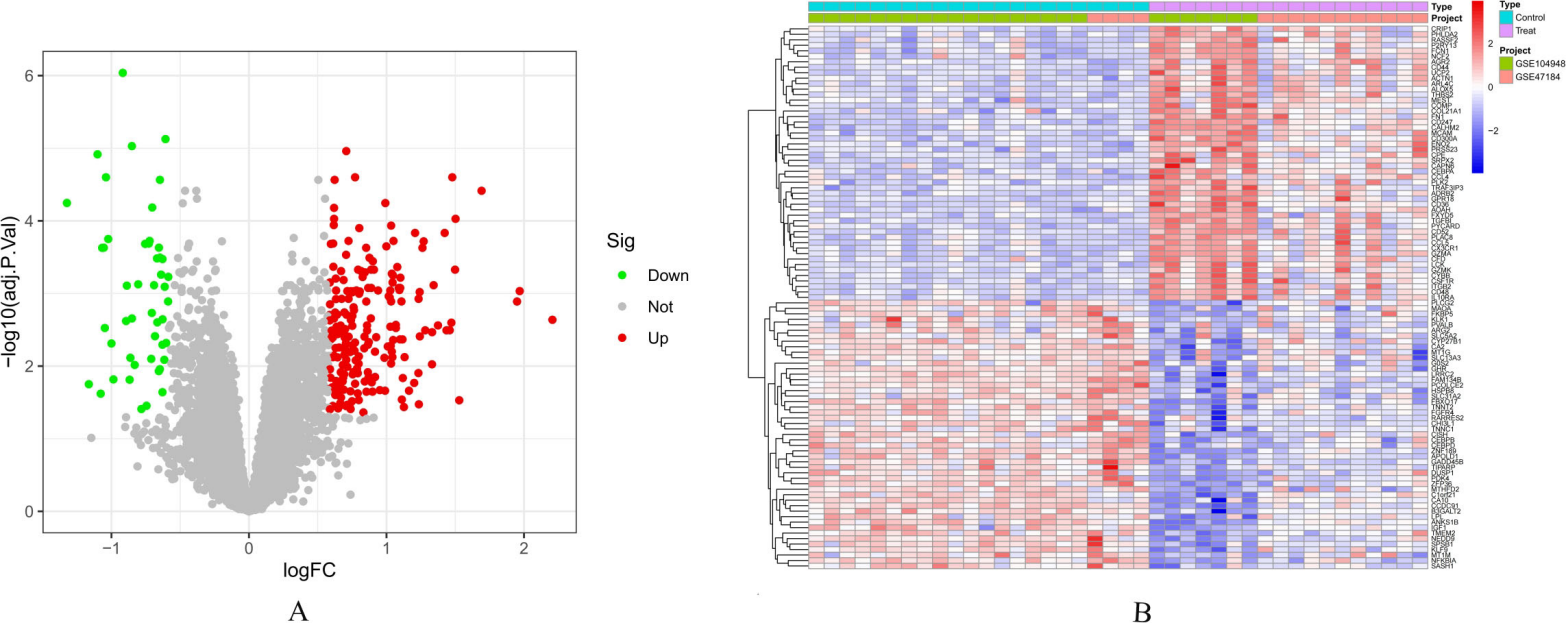
Identification of differentially expressed genes in diabetic nephropathy. **(A)** Volcano plot of diabetic nephropathy DEGs (red: upregulated genes; green: downregulated genes) **(B)** Heatmap of diabetic nephropathy DEG expression profiles.

Using thresholds of |logFC| > 0.585 and adjusted p-value < 0.05, we identified 259 DEGs in the diabetic nephropathy dataset, including 210 upregulated and 49 downregulated genes (**Figure 3**).

### 3.3 Construction of weighted gene co-expression networks and module identification

**Figure 4:**
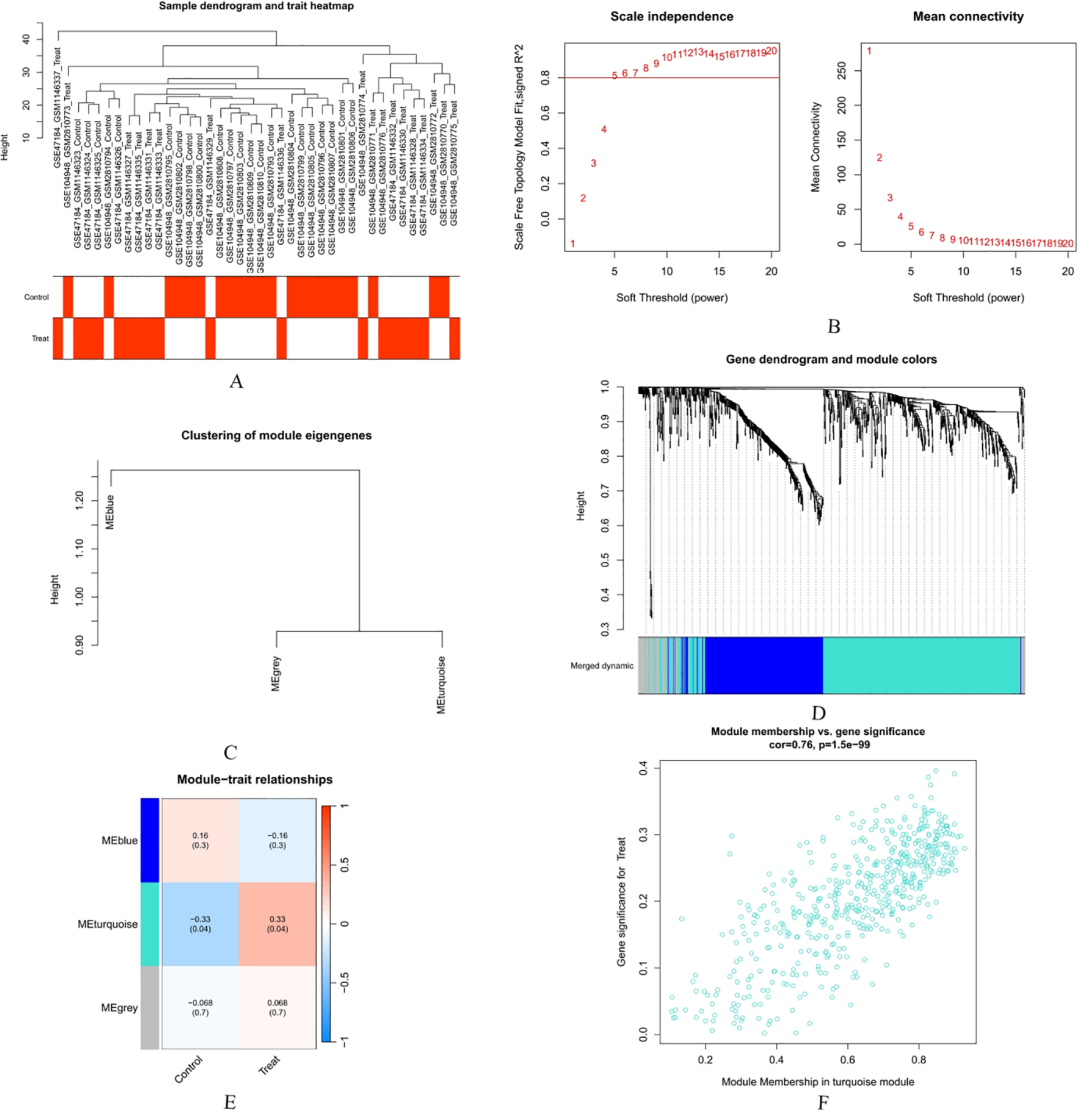
Establishment and analysis of the weighted gene co-expression network: **(A)** Display of clustering and phenotype in the combined data samples; the top part of the figure represents clustering, while the bottom part depicts phenotype. Colors indicate the status of the disease. A red square indicates that the correlation with the disease or control group in the sample is 1; a white square indicates a correlation of 0. **(B)** Determination of a soft threshold. **(C)** Integration of similar modules using the dynamic tree-cutting algorithm. **(D)** Genes with varying degrees of similarity are clustered in a dendrogram based on topological overlap and the designated module color. **(E)** A heat map showcases the correlation between different modules and clinical features. The vertical axis represents various modules, while the horizontal axis indicates different traits. Each small block displays the correlation coefficient and its P-value between the module and the trait. **(F)** In the turquoise modules, the relationship between module members and gene significance is explored. “Con” stands for the control group.

To ensure a scale-free network, we set the soft threshold to a β value of 10 (scale-free R² = 0.9). Genes with similar expression patterns were clustered into co-expression modules and labeled with different colors, resulting in the identification of 3 modules. The module-trait relationships revealed the associations between each module and clinical information. The turquoise module (correlation coefficient = 0.33, p = 0.04) was significantly correlated with depression and was therefore selected for further analysis, comprising a total of 523 genes. High intramodular connectivity, as measured by GS and MM, is a characteristic feature of module genes in co-expression networks. The scatter plot of GS (y-axis) versus MM (x-axis) is displayed in the figure in blue and gray (**Figure 4**).

### 3.4 Download of PANoptosis genes and identification of intersecting genes

**Figure 5:**
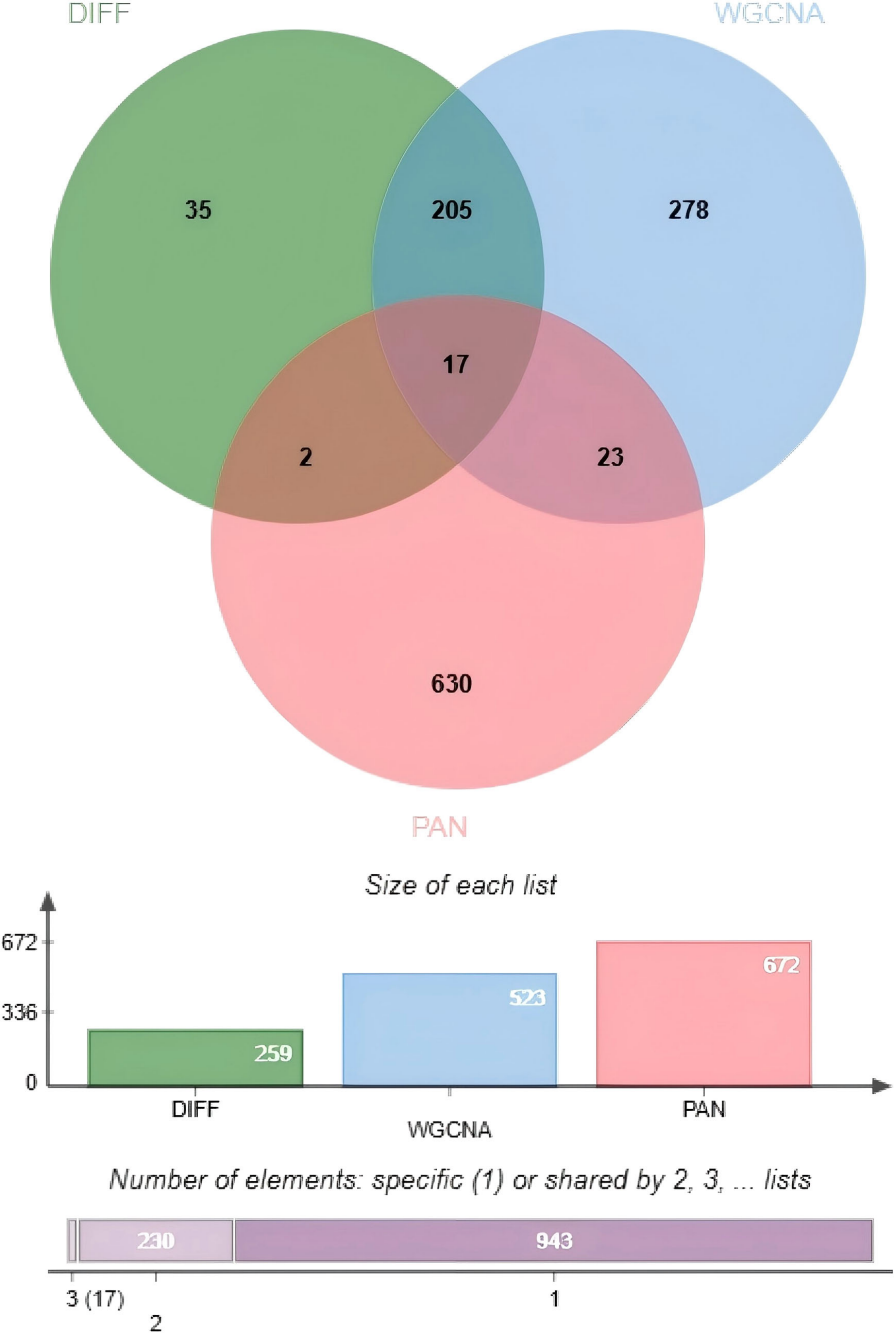
Venn diagram analysis of shared genes between diabetic nephropathy and PANoptosis.

A total of 726 pan-apoptotic genes were downloaded from the literature published by Li Li. Venn diagram analysis was performed on diabetic nephropathy, WGCNA key genes, and pan-apoptotic genes for intersection analysis, identifying 17 intersecting differentially expressed genes (**Figure 5**).

### 3.5 GO and KEGG enrichment analysis

**Figure 6:**
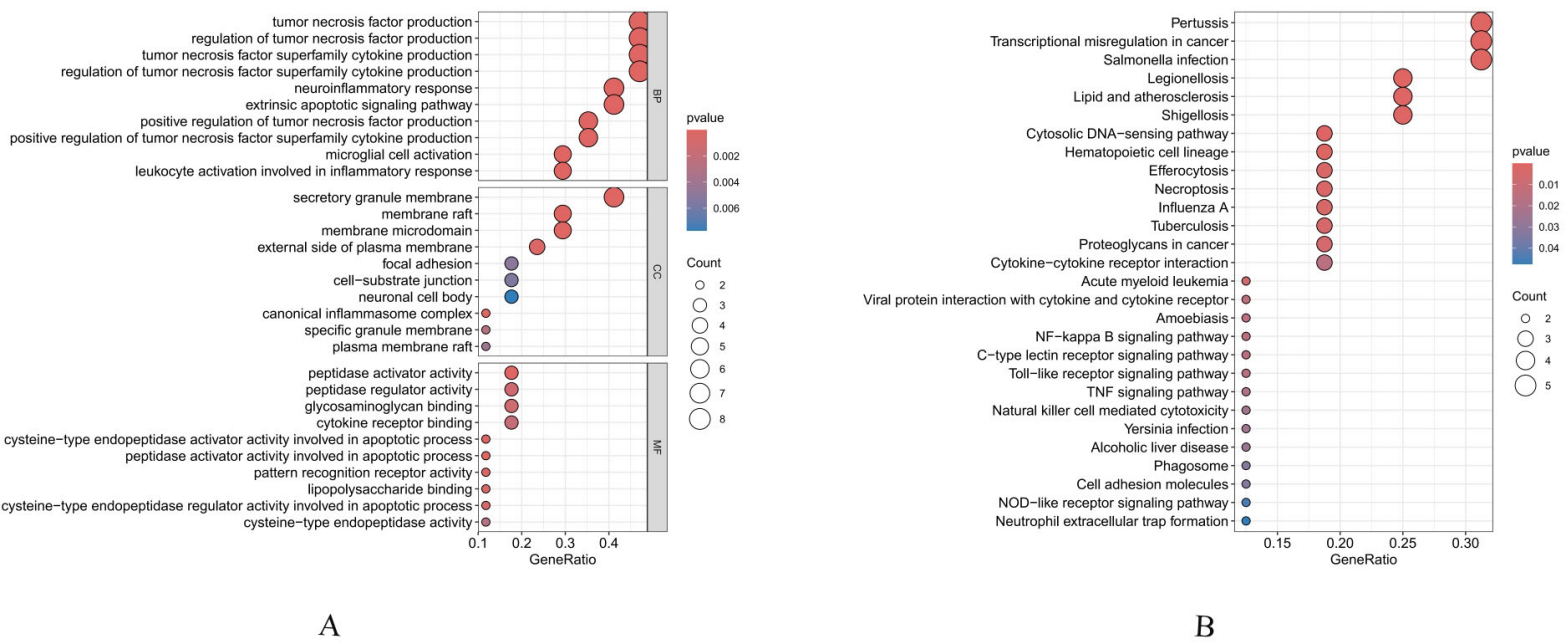
Functional enrichment analysis of intersecting genes. **(A)** Bubble plot of GO enrichment analysis. **(B)** Bubble plot of KEGG pathway enrichment analysis.

The “clusterProfiler” package in R was employed to conduct GO functional enrichment analysis and KEGG pathway analysis on the 17 intersecting genes. The Key findings from GO enrichment analysis revealed: Biological processes: neuroinflammatory response, tumor necrosis factor production, regulation of tumor necrosis factor production, tumor necrosis factor superfamily cytokine production, and regulation of tumor necrosis factor superfamily cytokine production. Molecular functions: peptidase activator activity, cysteine-type endopeptidase activator activity involved in apoptotic process, peptidase activator activity involved in apoptotic process, pattern recognition receptor activity, and lipopolysaccharide binding. Cellular components: secretory granule membrane, membrane raft, membrane microdomain, canonical inflammasome complex, and external side of plasma membrane (**Figure 6A**). KEGG pathway analysis demonstrated significant enrichment in: Pertussis, Lipid and atherosclerosis, NF-kappa B signaling pathway, Toll-like receptor signaling pathway, TNF signaling pathway (**Figure 6B**).

### 3.6 Identification of optimal feature genes using machine learning

**Figure 7:**
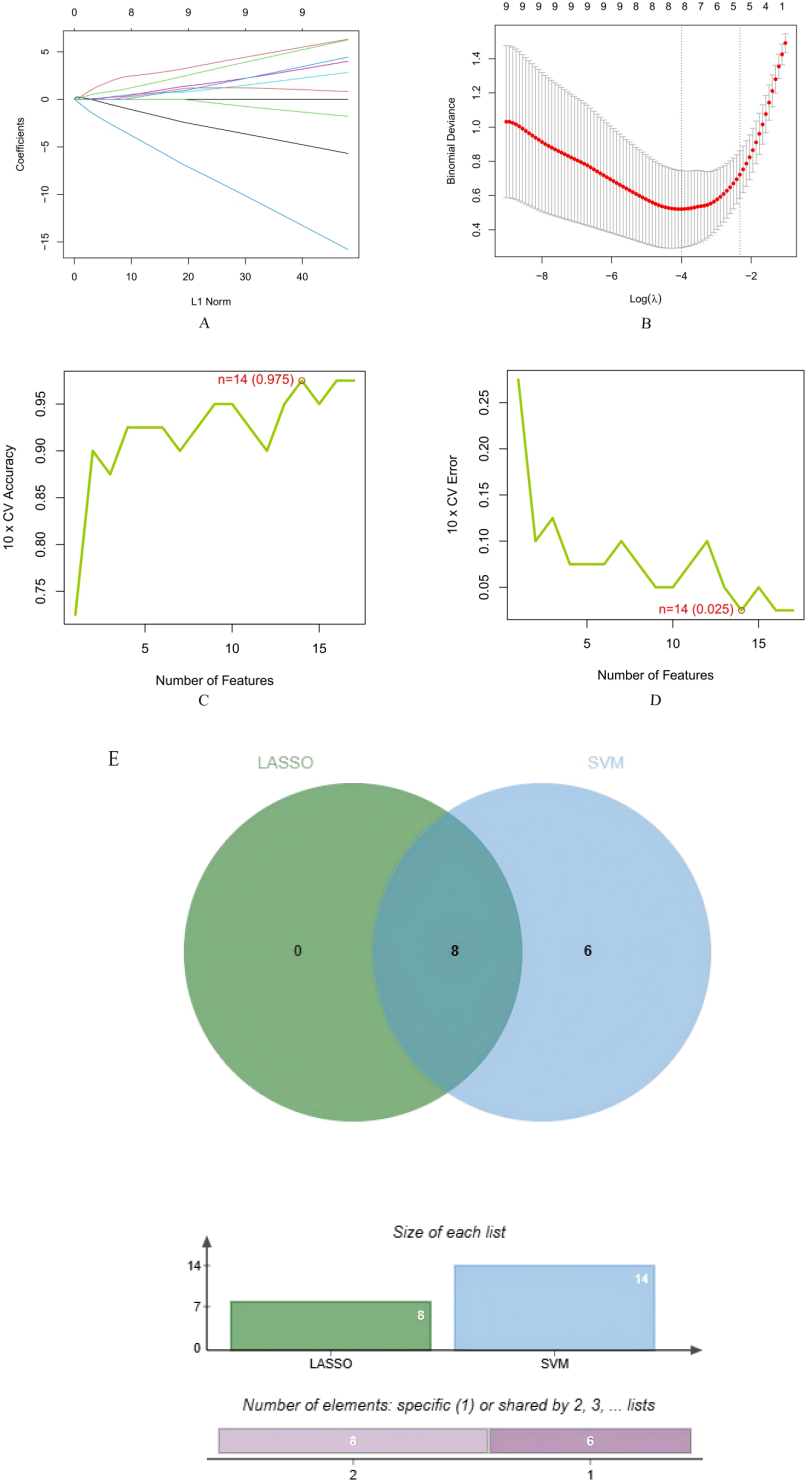
Machine learning results for diabetic nephropathy; **(A, B)** LASSO regression analysis; **(C, D)** Support Vector Machine-Recursive Feature Elimination (SVM-RFE); **(E)** Consensus genes identified by both machine learning methods.

Based on the diabetic nephropathy differential gene expression matrix data, we employed two machine learning approaches to identify potential biomarkers. LASSO regression analysis identified 8 candidate DN biomarkers: CD44, CRIP1, CEBPB, TNFRSF1B, CAV1, IGF1, GZMB, and LY96.SVM-RFE algorithm selected 15 potential biomarkers: CEBPB, CRIP1, IGF1, CD44, CX3CR1, LY96, CAV1, TNFRSF1B, PTPRC, TYROBP, ITGAM, GZMB, PYCARD, and SFRP1.The intersection of results from both machine learning methods yielded 8 consensus genes:CD44, CRIP1, CEBPB, TNFRSF1B, CAV1, IGF1, GZMB, and LY96 (**Figure 7**).

### 3.7 Expression of hub genes and validation in external datasets

**Figure 8:**
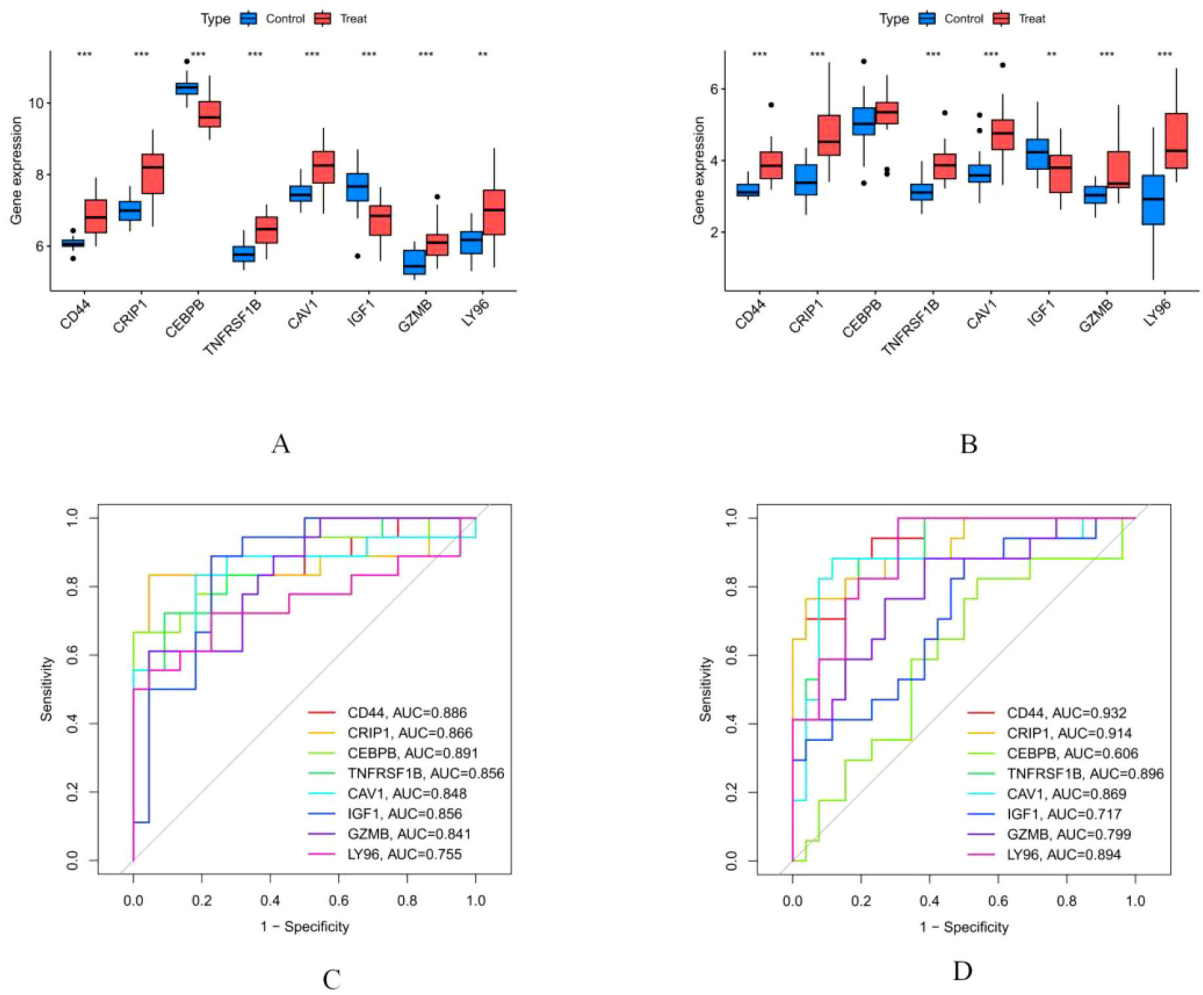
Expression of Hub Genes and Validation in External Datasets. **(A)** Expression of hub genes in the training set. ** p < 0.01; *** p < 0.001; CAV1 p=0.0001, CD44 p=3.11e-05, CEBPB p=1.7e-06, CRIP1 p=2.67e-05, GZMB p=0.0001, IGF1 p=8.75e-05, LY96 p=0.0030, TNFRSF10B p=1.94e-05; **(B)** Expression of hub genes in the validation set: CAV1 p=1.67e-05, CD44 p=1.32e-07, CEBPB p=0.4768, CRIP1 p=3.4e-05, GZMB p=0.0007, IGF1 p=0.0097, LY96 p=3.96e-06, TNFRSF10B p=4.88e-06; **(C)** ROC curve of the diabetic nephropathy training set; **(D)** ROC curve of the diabetic nephropathy validation set.

Compared to normal control samples, we found that CD44, CRIP1, TNFRSF1B, CAV1, IGF1, GZMB, and LY96 were expressed at higher levels in the DN group in the training set, while CEBPB was downregulated. Subsequently, we validated the expression of these genes in an independent validation set. The results showed that CD44, CRIP1, CEBPB, TNFRSF1B, CAV1, GZMB, and LY96 were significantly upregulated in the DN group, whereas IGF1 was downregulated. All differences were statistically significant (**Figures 8A, B**).

### 3.8 ROC curve analysis

To evaluate the comprehensive diagnostic performance of the core gene panel in both training and validation sets, we constructed receiver operating characteristic (ROC) curves. The results demonstrated: In the training set, the AUC values for CD44, CRIP1, CEBPB, TNFRSF1B, CAV1, IGF1, GZMB, and LY96 were 0.886, 0.866, 0.891, 0.856, 0.848, 0.856, 0.841, and 0.755, respectively. In the validation set, the predictive performance remained stable, with AUC values of 0.932, 0.914, 0.606, 0.896, 0.869, 0.717, 0.799, and 0.894, respectively. The sensitivity, specificity, positive and negative predictive values of were for training set and validation set were listed in **Table 2**, **Table 3**, respectively. These results indicate that this gene panel exhibits excellent disease-discriminatory ability and could serve as potential diagnostic biomarkers for diabetic nephropathy (**Figures 8C, D**).

**Table 2:** ROC curves performance for training set.

Gene	AUC	Cutoff	Sensitivity	Specificity	PPV	NPV
CD44	0.886(0.765-0.992)	6.329	0.833	0.954	0.938	0.875
CRIP1	0.866(0.715-0.992)	7.387	0.833	0.955	0.938	0.875
CEBPB	0.891(0.758-0.980)	9.866	0.667	1	1	0.786
TNFRSF1B	0.856(0.715-0.955)	6.309	0.722	0.909	0.867	0.8
CAV1	0.848(0.699-0.962)	7.714	0.833	0.818	0.789	0.857
IGF1	0.856(0.720-0.957)	7.240	0.889	0.773	0.762	0.895
GZMB	0.841(0.702-0.942)	6.019	0.611	0.9554	0.917	0.75
LY96	0.755(0.571-0.919)	6.749	0.556	0.955	0.909	0.724

**Table 3:** ROC curves performance for validation set.

Gene	AUC	Cutoff	Sensitivity	Specificity	PPV	NPV
CD44	0.932(0.851-0.986)	3.366	0.941	0.769	0.727	0.952
CRIP1	0.914(0.812-0.986)	4.148	0.765	0.962	0.929	0.862
TNFRSF1B	0.869(0.731-0.982)	3.957	0.882	0.885	0.833	0.92
CAV1	0.717(0.550-0.867)	4.270	0.882	0.5	0.536	0.8667
IGF1	0.799(0.649-0.923)	3.113	0.882	0.615	0.6	0.889
GZMB	0.894(0.792-0.966)	3.300	1	0.692	0.68	1
LY96	0.932(0.851-0.986)	3.366	0.941	0.769	0.727	0.952

### 3.9 GSEA enrichment analysis

The GSEA results demonstrated that in diabetic nephropathy, the hub genes were collectively enriched in the following KEGG pathways:

NATURAL_KILLER_CELL_MEDIATED_CYTOTOXICITY, INTESTINAL_IMMUNE_NETWORK_FOR_IGA_PRODUCTION, CYTOKINE_CYTOKINE_RECEPTOR_INTERACTION, CHEMOKINE_SIGNALING_PATHWAY, PPAR_SIGNALING_PATHWAY,FATTY_ACID_METABOLISM,and ECM_RECEPTOR_INTERACTION (**Supplementary Figure 1**).

### 3.10 GSVA enrichment analysis

The GSVA analysis results revealed that the core genes were collectively involved in 94 signaling pathways in patients with diabetic nephropathy. These pathways primarily included: KEGG_JAK_STAT_SIGNALING_PATHWAY, KEGG_P53_SIGNALING_PATHWAY, KEGG_PPAR_SIGNALING_PATHWAY, Additionally, pathways related to immunity, inflammation, and metabolism were significantly enriched (**Supplementary Figure 2**).

### 3.11 Immune infiltration analysis

To investigate the pathogenesis of diabetic nephropathy (DN), we analyzed immune cell infiltration patterns in patient tissues using the CIBERSORT algorithm to quantify infiltration scores for various immune cell types. Key findings from the DN dataset revealed significant alterations compared to normal controls: Decreased infiltration: T regulatory cells (Tregs), activated mast cells, and neutrophils Increased infiltration: Gamma delta T cells (Tγδ) and resting mast cells.

**Figure 9:**
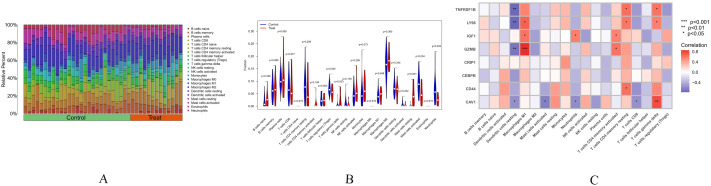
**(A)** Immune cell composition in diabetic nephropathy analyzed using the CIBERSORT algorithm. **(B)** Differential analysis of immune cell components in the diabetic nephropathy dataset. **(C)** Correlation analysis between immune cell composition and hub gene expression.

Correlation analysis between core genes and immune cells demonstrated strong associations with: Dendritic cells (resting), M1 macrophages, activated mast cells, neutrophils, CD4+ memory T cells (activated/resting), CD8+ T cells, and Tγδ cells (**Figure 9**).

### 3.12 Potential drug prediction and molecular docking analysis

Based on the hub genes identified by the machine learning model, we performed potential drug prediction using the DsigDB database from the Enrichr platform. The DsigDB analysis revealed that several drugs, including imatinib, sodium nitroprusside, GNF-Pf-4325, pyrrolidine dithiocarbamate, and actinomycin D, may have therapeutic potential for diabetic nephropathy. Based on our prediction, imatinib emerged as the most significantly enriched drug.

**Figure 10:**
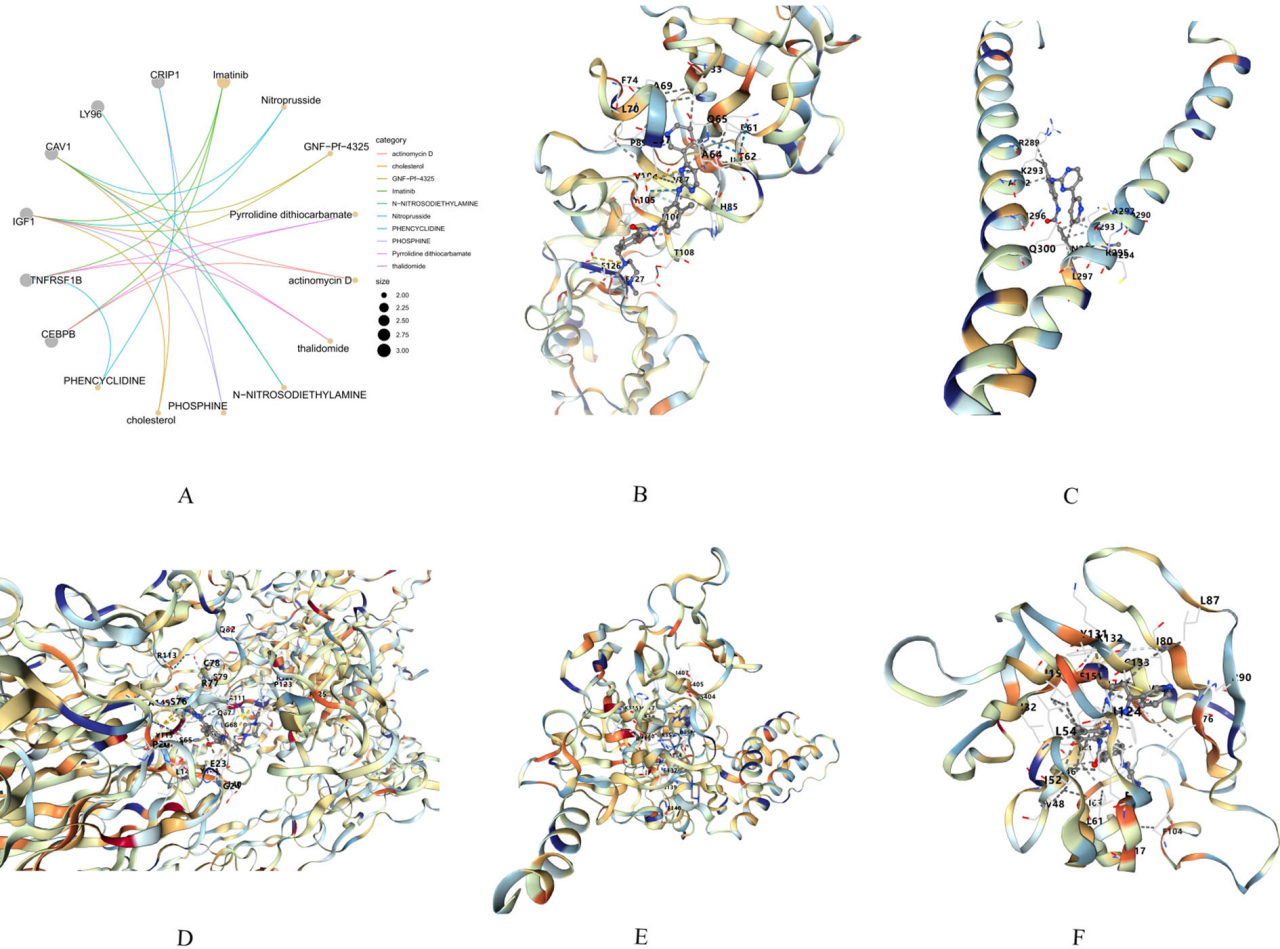
**(A)** Drug prediction. **(B–F)** Molecular docking of imatinib with hub genes.: CD44, CEBPB, TNFRSF1B, CAV1, LY96.

To further validate its potential, we conducted molecular docking between imatinib and the hub genes (CD44, CEBPB, TNFRSF1B, CAV1, LY96). Generally, a lower binding energy indicates more stable binding activity between the compound and the target protein: Binding energy < 0 kcal/mol suggests binding affinity. Binding energy < -5.0 kcal/mol indicates strong binding capability. Binding energy < -7.5 kcal/mol reflects extremely high binding affinity. The molecular docking results demonstrated that imatinib exhibited strong binding activity with all tested hub genes:CD44: -8.2 kcal/mol, CEBPB: -7.5 kcal/mol, TNFRSF1B: -10.3 kcal/mol, CAV1: -8.0 kcal/mol, LY96: -10.3 kcal/mol, Notably, all binding energies were negative and below -6 kcal/mol, confirming robust interactions between imatinib and the hub genes. These findings suggest that imatinib may have the capability to target the key molecular pathways in the pathogenesis and development of DN (**Figure 10**).

### 3.13 Single-nucleus RNA sequencing

**Figure 11:**
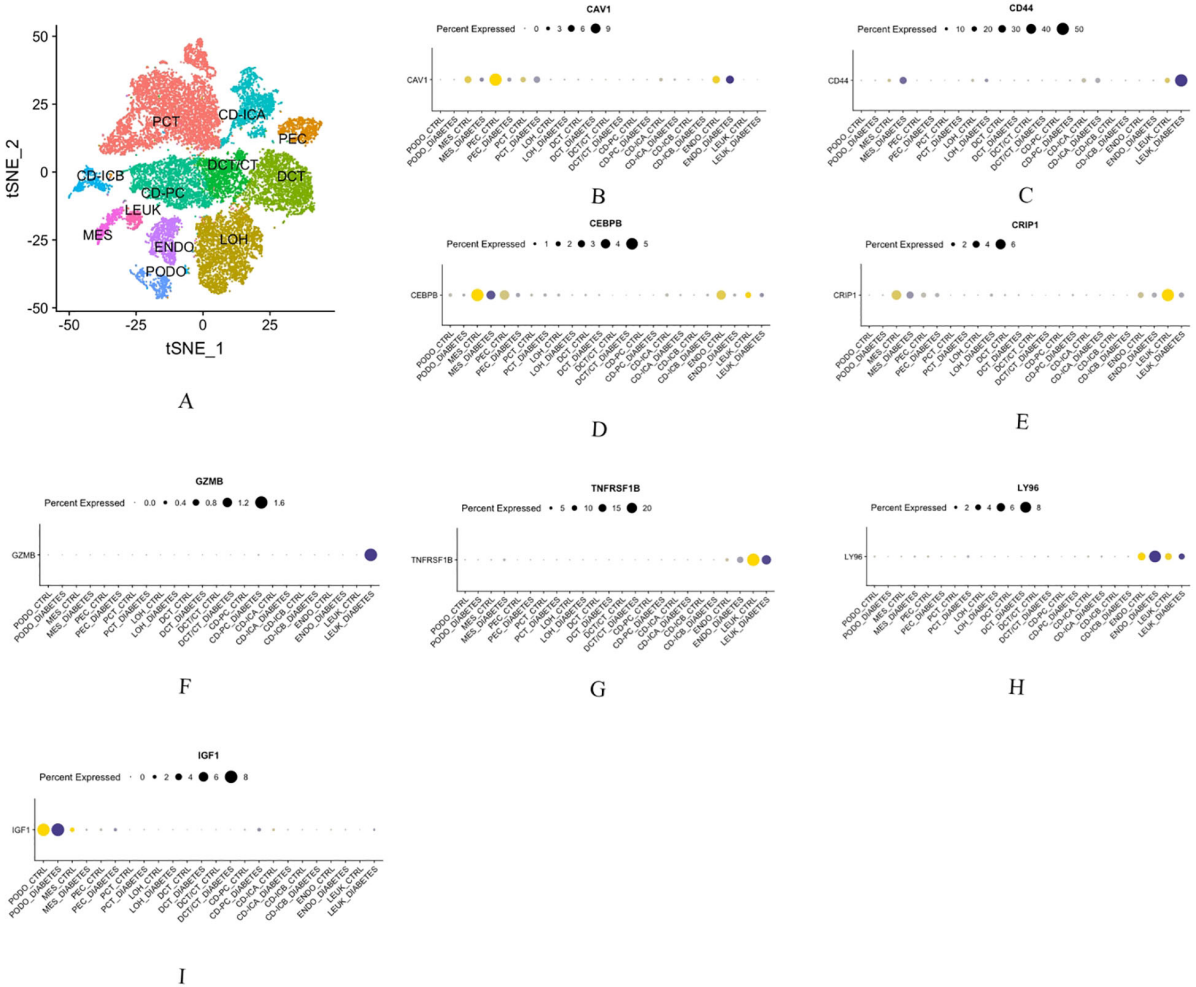
Single Nucleus RNA Sequencing **(A)** The distribution of hub genes in 12 cell groups. **(B)** CAV1. **(C)** CD44. **(D)** CEBPB. **(E)** CRIP1. **(F)** GZMB. **(G)** TNFRSF1B. **(H)** LY96. **(I)** IGF1.

Single-nucleus RNA sequencing analysis revealed the cellular distribution patterns of hub genes across 12 distinct cell populations. Specifically: IGF1 exhibited predominant expression in podocytes, LY96 was primarily localized to endothelial cells, CD44, CRIP1, GZMB, and TNFRSF1B showed main distribution in leukocytes, CAV1 was chiefly expressed in glomerular parietal epithelial cells CEBPB demonstrated dominant expression in mesenchymal stem cells (**Figure 11**).

### 3.14 Construction of the core gene ceRNA network

**Figure 12:**
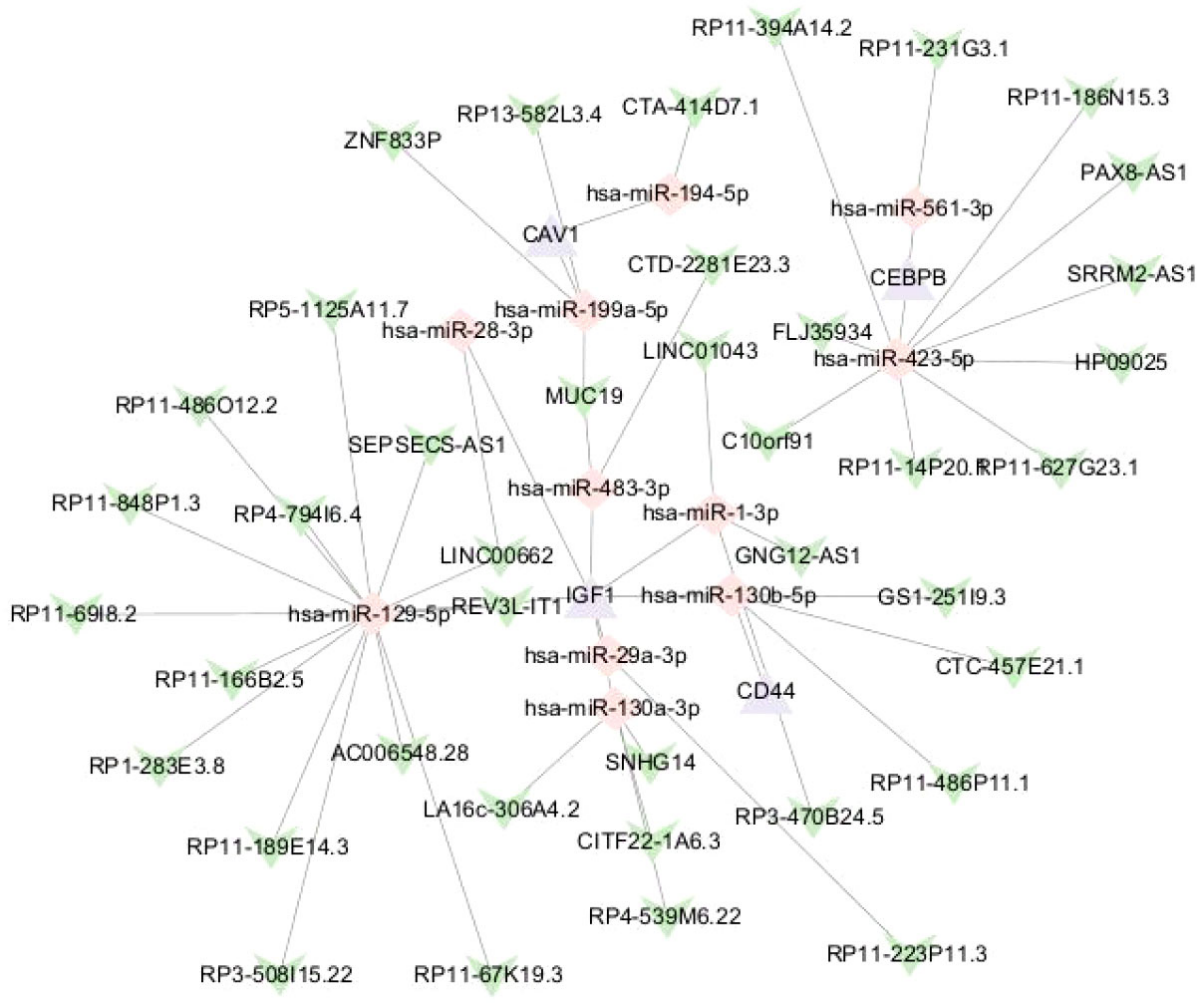
Construction of the ceRNA network.

Using the miRanda, miRDB, and TargetScan databases, we predicted miRNAs associated with CD44, CRIP1, CEBPB, TNFRSF1B, CAV1, IGF1, GZMB, and LY96, yielding 34 mRNA-miRNA interaction pairs. Subsequently, the spongeScan database was employed to retrieve lncRNAs corresponding to these 34 miRNAs, resulting in 42 miRNA-lncRNA interaction pairs. Finally, Cytoscape 3.9.1 was utilized to construct a ceRNA regulatory network comprising 55 nodes (11 miRNAs, 4 mRNAs, and 40 lncRNAs) and 54 edges (**Figure 12**).

### 3.15 qRT-PCR results

**Figure 13:**
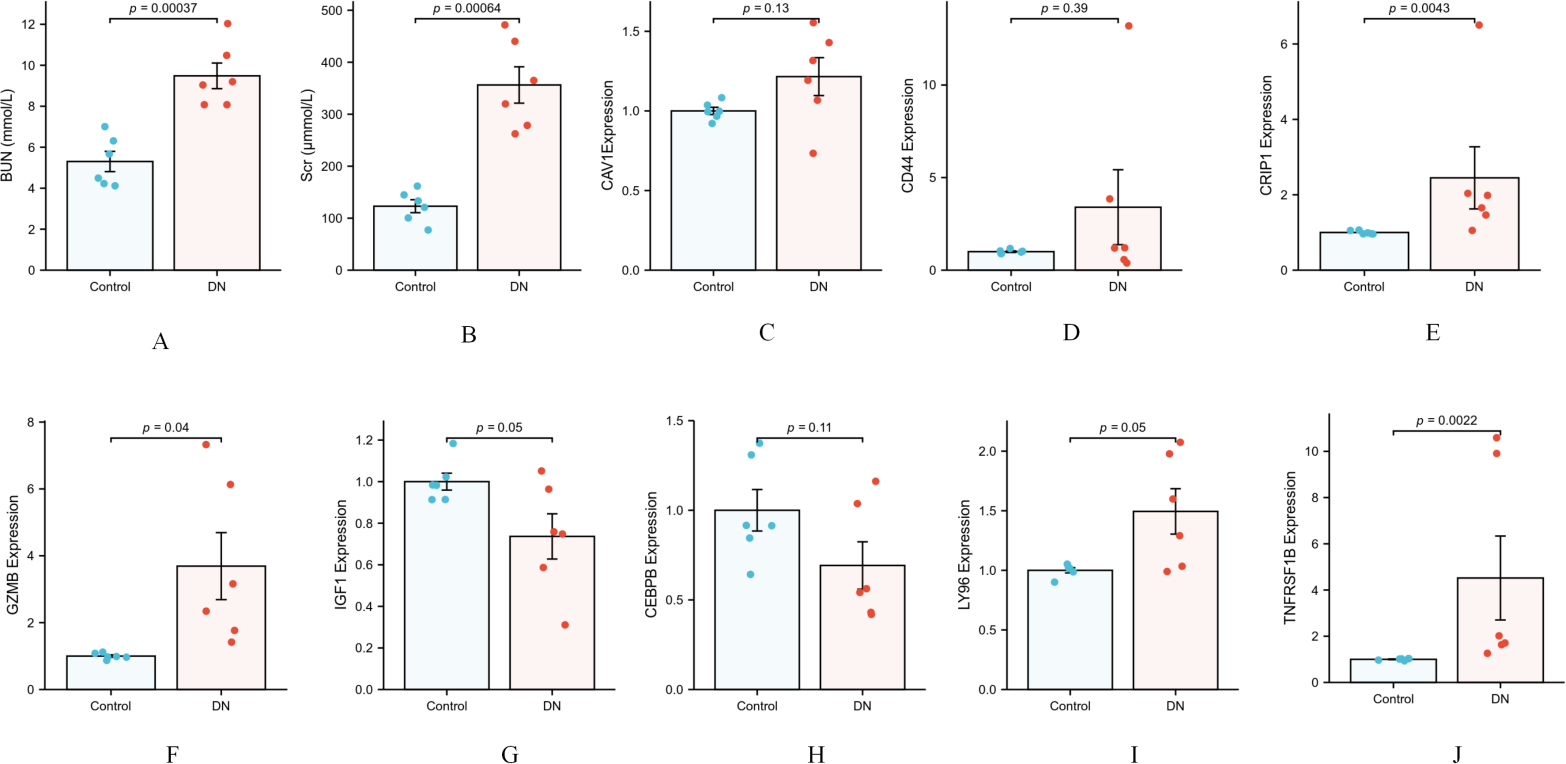
**(A)** Comparison of serum creatinine in mice; **(B)** comparison of blood urea nitrogen in mice; **(C–J)** mRNA expression of hub genes by RT-qPCR.

We employed qRT-PCR to examine the expression levels of PANoptosis-related genes in renal tissues from both control and diabetic nephropathy (DN) groups. The results demonstrated statistically significant differences (P < 0.05) in the expression of five genes: GZMB, CRIP1, LY96, TNFRSF1B, and IGF1. Specifically: Upregulated in DN group: TNFRSF1B, GZMB, CRIP1, and LY96 showed significantly higher expression levels compared to controls. Downregulated in DN group: IGF1 expression was markedly reduced. No significant difference: CAV1, CEBPB, and CD44 exhibited comparable expression levels between the two groups (**Figure 13**).

## 4 Discussion

The global prevalence of diabetes continues to rise, with an estimated 700 million adults aged 20–79 projected to be affected by 2045. Among them, approximately 20–40% of diabetic patients will develop DN within 10 years of disease onset, making DN a significant public health challenge worldwide. Consequently, preventing and delaying DN progression has become a critical focus in modern medical research (23, 24).

In this study, through bioinformatics analysis of DN datasets from the GEO database, we identified 17 differentially expressed PANoptosis-related genes associated with DN. GO enrichment analysis revealed that these genes are primarily involved in biological processes such as inflammation and immune responses, cell death and survival, metabolism and stress responses, and cell proliferation and differentiation. KEGG pathway analysis demonstrated significant enrichment in the NF-κB signaling pathway, Toll-like receptor signaling pathway, necroptosis, and TNF signaling pathway in DN.

Using LASSO and SVM-RFE machine learning algorithms, we screened eight core genes: CD44, CRIP1, TNFRSF1B, CAV1, IGF1, GZMB, LY96, and CEBPB, which were further validated in external datasets. Three of the eight proposed biomarkers, CD44, CEBPB, and CAV1, show no significant difference in the qRT-PCR validation. Receiver operating characteristic (ROC) curve analysis confirmed the excellent diagnostic performance of these genes. TNFRSF1B, a key member of the tumor necrosis factor receptor superfamily, regulates inflammation, immune responses, stress responses, host defense, and apoptosis. Elevated circulating soluble TNFRSF1B levels are associated with DN and independently predict cardiovascular events and mortality risk, even beyond microalbuminuria and renal function (25). Angiopoietin-2 (ANGPT2), a member of the endothelial growth factor family, promotes albumin transcytosis in glomerular endothelial cells (GECs) under high glucose conditions by upregulating CAV1 expression, thereby increasing albuminuria (26). IGF1, a polypeptide hormone, contributes to DN pathogenesis by binding to the IGF1 receptor (IGF1R) to regulate growth, differentiation, and insulin metabolism. Renal IGF1 expression is markedly increased in DN (27, 28). GZMB, LY96, and CRIP1 have not been previously reported in DN, warranting further investigation. qRT-PCR validation confirmed the bioinformatics predictions for these core genes.

The pathogenesis of DN is complex, with emerging evidence highlighting the critical role of immune mechanisms (29, 30). Our study revealed: Decreased levels: T regulatory cells (Tregs), activated mast cells, and neutrophils in DN samples. Tregs, a specialized T-cell subset, maintain peripheral tolerance and immune homeostasis. Their downregulation in DN (correlated negatively with pyroptosis, r = −0.4191, P = 0.0522) suggests a protective role (31).Increased levels: Gamma delta T cells (Tγδ) and resting mast cells. Mast cells, innate immune cells derived from bone marrow, are enriched in diabetic kidneys and promote renal fibrosis via TGF-β1 and protease release (32). Neutrophils, though abundant in acute kidney injury (AKI), exhibit controversial roles in DN. While elevated neutrophil-to-lymphocyte ratios correlate with microalbuminuria and declining GFR, direct evidence linking neutrophil depletion to DN progression remains lacking (32).

Immune correlation analysis showed that the eight core genes predominantly interact with dendritic cells (resting), M1 macrophages, activated mast cells, neutrophils, CD4+ memory T cells (activated/resting), CD8+ T cells, and Tγδ cells, implicating their involvement in DN’s immune microenvironment.

Results from drug screening suggest that imatinib may have the capability to target the key molecular pathways in the pathogenesis and development of DN. Experimental studies demonstrate that imatinib ameliorates diabetes-induced renal dysfunction and structural damage by suppressing overexpression of growth factors, collagen, proliferating cells, α-smooth muscle actin-positive cells, and macrophage infiltration (33). However, whether imatinib can target these molecules *in vivo* and whether it can exert therapeutic effect against DN needs further exploration.

### 4.1 Limitations

Limitations of this study include reliance on publicly available datasets, potential selection bias in gene identification, small size of sample size in the *in vivo* studies, limited generalizability to different populations, cross-sectional data analysis, and the need for further experimental verification to elucidate the functional mechanisms of the identified genes. Additionally, this study identified CD44, CRIP1, CEBPB, TNFRSF1B, CAV1, IGF1, GZMB and LY96 as potential diagnostic biomarkers for DN using comprehensive bioinformatics and machine learning methods. Three of the eight proposed biomarkers, CD44, CEBPB, and CAV1, show no significant difference in the qRT-PCR validation. This discrepancies showed that when it comes to human beings, all the biomolecules function in a very complex biomatrix, which led to the discrepancies between theoretical predicted biomarkers and biomarkers identified using qRT-PCR. In addition, for the machine learning approach, the training set is also very important. Inadequate training might also be a reasonable explanation for the discrepancies. All the results in this present study provide a basis for potential peripheral blood diagnostic markers, but additional validation and clinical translation are required for practical application in clinical settings. Techniques such as real-time quantitative PCR or immunohistochemistry are used to examine the expression levels of these candidate genes in clinical samples with bigger sample size. Differences in gene expression between patients with DN and healthy controls needs to be compared. Further statistical methods are used to determine whether there is a significant correlation and to evaluate the feasibility of these genes as potential biomarkers.

## 5 Conclusions

This study integrates multi-omics analyses to elucidate PANoptosis-related mechanisms in DN, identifying five diagnostic biomarkers with validated immune correlations. Imatinib was identified to matinib may have the capability to target the key molecular pathways in the pathogenesis and development of DN. Future studies should explore the translational potential of these findings. For example, the effects of these genes on the development and progression of DN can be investigated through gene knockout or overexpression. Clinical cohort studies are needs to be conducted to track the disease progression and treatment response of patients with DN, and to evaluate the potential of these candidate genes as predictive or prognostic markers. Drug intervention trials are conducted to assess the efficacy of pharmacological treatment targeting these genes in DN patients.

## Data Availability

The original contributions presented in the study are included in the article/**Supplementary Material**. Further inquiries can be directed to the corresponding authors.

## Glossary

DN: diabetic nephropathy
DEGs: differentially expressed genes
WGCNA: weighted gene co-expression network analysis
AUC: area under the curve
CIBERSORT: Cell-type Identification By Estimating Relative Subsets Of RNA Transcripts
DSigDB: Disease Signature Database
GO: Gene Ontology
KEGG: Kyoto Encyclopedia of Genes and Genomes
LASSO: least absolute shrinkage and selection operator
SVM: support vector machine
ESRD: end-stage renal disease
CKD: chronic kidney disease
ACEIs: angiotensin-converting enzyme inhibitors
ARBs: angiotensin II receptor blockers
RAAS: renin-angiotensin-aldosterone system
GSDMD: Gasdermin D
TLR4/NF-κB: Toll-like receptor 4/Nuclear factor kappa-B
DR5: receptor Death receptor 5
NCBI: National Center for Biotechnology Information
WGCNA: Weighted gene co-expression network analysis
TOM: Topological overlap matrix
MEs: Module eigengenes
MM: Module membership
GS: Gene significance
BP: Biological processes
MF: Molecular functions
CC: Cellular components
ROC: Receiver operating characteristic
GSEA: Gene set enrichment analysis
GSVA: Gene set variation analysis
ceRNACompeting: endogenous RNA
miRNA: micro RNA
lncRNA: long non-coding RNA
BUN: Blood urea nitrogen
CREA: Serum creatinine
PCA: Principal component analysis
TNF: Tumor Necrosis Factor
CD44: Cluster-of-differentiation gene 44
CRIP1: Cysteine-Rich Protein 1
CEBPB: CCAAT-Enhancer-Binding Protein Beta
TNFRSF1B: Tumor Necrosis Factor Receptor Superfamily Member 1B
CAV1: Caveolin-1
IGF1: Insulin-Like Growth Factor 1
GZMB: Granzyme B
LY96: Lymphocyte Antigen 96
CX3CR1: C-X3-C Motif Chemokine Receptor 1
PTPRC: Protein Tyrosine Phosphatase Receptor Type C
TYROBP: TYrosine Kinase-Binding Protein
ITGAM: Integrin Subunit Alpha M
GZMB: Granzyme B
PYCARD: PYD and CARD domain containing protein
SFRP1: Secreted frizzled related protein 1
Tregs: T regulatory cells
Tγδ: Gamma delta T cells
ANGPT2: Angiopoietin-2
GECs: Glomerular endothelial cells
IGF1R: Insulin-like growth factor I receptor
AKI: Acute kidney injury
GFR: Glomerular filtration rate.

## References

[1] Handelman GS Kok HK Chandra RV Razavi AH Lee MJ Asadi H . eDoctor: machine learning and the future of medicine. J Intern Med. (2018) 284:603–19. doi: 10.1111/joim.12822, PMID: 30102808

[2] Saeedi P Petersohn I Salpea P Malanda B Karuranga S Unwin N . Global and regional diabetes prevalence estimates for 2019 and projections for 2030 and 2045: Results from the International Diabetes Federation Diabetes Atlas, 9th edition. Diabetes Res Clin Pract. (2019) 157:107843. doi: 10.1016/j.diabres.2019.107843, PMID: 31518657

[3] Mayer B . Using systems biology to evaluate targets and mechanism of action of drugs for diabetes comorbidities. Diabetologia. (2016) 59:2503–6. doi: 10.1007/s00125-016-4032-2, PMID: 27376542

[4] Eddy S Mariani LH Kretzler M . Integrated multi-omics approaches to improve classification of chronic kidney disease. Nat Rev Nephrol. (2020) 16:657–68. doi: 10.1038/s41581-020-0286-5, PMID: 32424281

[5] Liu F Fu Y Wei C Chen Y Ma S Xu W . The expression of GPR109A, NF-kB and IL-1β in peripheral blood leukocytes from patients with type 2 diabetes. Ann Clin Lab Sci. (2014) 44:443–8. doi: 10.1007/s40268-020-00334-z, PMID: 25361930

[6] Ojima A Matsui T Nishino Y Nakamura N Yamagishi S . Empagliflozin, an inhibitor of sodium-glucose cotransporter 2 exerts anti-inflammatory and antifibrotic effects on experimental diabetic nephropathy partly by suppressing AGEs-receptor axis. Horm Metab Res. (2015) 47:686–92. doi: 10.1055/s-0034-1395609, PMID: 25611208

[7] Bernardi S Michelli A Zuolo G Candido R Fabris B . Update on RAAS modulation for the treatment of diabetic cardiovascular disease. J Diabetes Res. (2016) 2016:8917578. doi: 10.1155/2016/8917578, PMID: 27652272 PMC5019930

[8] Tanaka Y Kume S Kitada M Kanasaki K Uzu T Maegawa H . Autophagy as a therapeutic target in diabetic nephropathy. Exp Diabetes Res. (2012) 2012:628978. doi: 10.1155/2012/628978, PMID: 22028701 PMC3199112

[9] Kume S Koya D . Autophagy: A novel therapeutic target for diabetic nephropathy. Diabetes Metab J. (2015) 39:451–60. doi: 10.4093/dmj.2015.39.6.451, PMID: 26706914 PMC4696980

[10] Brennan MA Cookson BT . Salmonella induces macrophage death by caspase-1-dependent necrosis. Mol Microbiol. (2000) 38:31–40. doi: 10.1046/j.1365-2958.2000.02103.x, PMID: 11029688

[11] Donate-Correa J Luis-Rodríguez D Martín-Núñez E Tagua VG Hernández-Carballo C Ferri C . Inflammatory targets in diabetic nephropathy. J Clin Med. (2020) 9:458. doi: 10.3390/jcm9020458, PMID: 32046074 PMC7074396

[12] Sun X Yang Y Meng X Li J Liu X Liu H . PANoptosis: mechanisms, biology, and role in disease. Immunol Rev. (2024) 321:246–62. doi: 10.1111/imr.13279, PMID: 37823450

[13] Gullett JM Tweedell RE Kanneganti T-D . ItD. all in the PAN: crosstalk, plasticity, redundancies, switches, and interconnectedness encompassed by PANoptosis underlying the totality of cell death-associated biological effects. Cells. (2022) 11:1495. doi: 10.3390/cells11091495, PMID: 35563804 PMC9105755

[14] Hou Y Lin S Qiu J Sun W Dong M Xiang Y . NLRP3 inflammasome negatively regulates podocyte autophagy in diabetic nephropathy. Biochem Biophys Res Commun. (2020) 521:791–8. doi: 10.1016/j.bbrc.2019.10.194, PMID: 31703838

[15] Sanz AB Sanchez-Niño MD Ramos AM Ortiz A . Regulated cell death pathways in kidney disease. Nat Rev Nephrol. (2023) 19:281–99. doi: 10.1038/s41581-023-00694-0, PMID: 36959481 PMC10035496

[16] Lv Z Hu J Su H Yu Q Lang Y Yang M . TRAIL induces podocyte PANoptosis via death receptor 5 in diabetic kidney disease. Kidney Int. (2025) 107:317–31. doi: 10.1016/j.kint.2024.10.026, PMID: 39571905

[17] Ju W Greene CS Eichinger F Nair V Hodgin JB Bitzer M . Defining cell-type specificity at the transcriptional level in human disease. Genome Res. (2013) 23:1862–73. doi: 10.1101/gr.155697.113, PMID: 23950145 PMC3814886

[18] Alakwaa F Das V Majumdar A Nair V Fermin D Dey AB . Leveraging complementary multi-omics data integration methods for mechanistic insights in kidney diseases. JCI Insight. (2025) 10:e186070. doi: 10.1172/jci.insight.186070, PMID: 40059827 PMC11949029

[19] Woroniecka KI Park AS Mohtat D Thomas DB Pullman JM Susztak K . Transcriptome analysis of human diabetic kidney disease. Diabetes. (2011) 60:2354–69. doi: 10.2337/db10-1181, PMID: 21752957 PMC3161334

[20] Martini S Nair V Keller BJ Eichinger F Hawkins JJ Randolph A . European renal cDNA bank; C-PROBE cohort; CKDGen consortium. Integr Biol identifies shared transcript Networks CKD J Am Soc Nephrol. (2014) 25:2559–72. doi: 10.1681/ASN.2013080906, PMID: 24925724 PMC4214523

[21] Tang J Yuan L Han Y-L . Metabolic-immune crosstalk in myocardial infarction: RLF and SMCHD1 identified as causal therapeutic targets via integrated lactylation-MR analysis.Front. Cell Dev Biol. (2025) 13:1653551. doi: 10.3389/fcell.2025.1653551, PMID: 41158309 PMC12554677

[22] Li L Juaiti M . Identification of a PANoptosis-related gene signature reveals therapeutic potential of SFRP2 in pulmonary arterial hypertension. Front Cardiovasc Med. (2025) 12:1521087. doi: 10.3389/fcvm.2025.1521087, PMID: 40364828 PMC12069314

[23] Zhang L Sun Z Yuan Y Sheng J . Integrating bioinformatics and machine learning to identify glomerular injury genes and predict drug targets in diabetic nephropathy. Sci Rep. (2025) 15:16868. doi: 10.1038/s41598-025-01628-5, PMID: 40374840 PMC12081755

[24] Tao Y Liu Y Wang Z Tang L Zhang Y Zheng S . Lumican as a potential biomarker for diabetic nephropathy. Ren Fail. (2025) 47:2480245. doi: 10.1080/0886022X.2025.2480245, PMID: 40195568 PMC11983523

[25] Gómez-Banoy N Cuevas V Higuita A Aranzález LH Mockus I . Soluble tumor necrosis factor receptor 1 is associated with diminished estimated glomerular filtration rate in Colombian patients with type 2 diabetes. J Diabetes Complicat. (2016) 30:852–7. doi: 10.1016/j.jdiacomp.2016.03.015, PMID: 27068267

[26] Chen Y Li H Zhang D Gong Y Jiang H Sun H . ANGPT2/CAV1 regulates albumin transcytosis of glomerular endothelial cells under high glucose exposure and is impaired by losartan. Nefrolog (Engl Ed). (2024) 44:50–60. doi: 10.1016/j.nefroe.2022.11.028, PMID: 36842857

[27] Vasylyeva TL Ferry RJ Jr. Novel roles of the IGF-IGFBP axis in etiopathophysiology of diabetic nephropathy. Diabetes Res Clin Pract. (2007) 76:177–86. doi: 10.1016/j.diabres.2006.09.012, PMID: 17011663 PMC1892792

[28] Marie R Pedersen JN Bærlocher L Koprowska K Pødenphant M Sabatel C . Single-molecule DNA-mapping and whole-genome sequencing of individual cells. Proc Natl Acad Sci U S A. (2018) 115:11192–7. doi: 10.1073/pnas.1804194115, PMID: 30322920 PMC6217438

[29] Ma J Li YJ Chen X Kwan T Chadban SJ Wu H . Interleukin 17A promotes diabetic kidney injury. Sci Rep. (2019) 9:2264. doi: 10.1038/s41598-019-38811-4, PMID: 30783187 PMC6381173

[30] Navarro JF Mora C Muros M García J . Urinary tumour necrosis factor-alpha excretion independently correlates with clinical markers of glomerular and tubulointerstitial injury in type 2 diabetic patients. Nephrol Dial Transplant. (2006) 21:3428–34. doi: 10.1093/ndt/gfl469, PMID: 16935891

[31] Jerome JR Deliyanti D Suphapimol V Kolkhof P Wilkinson-Berka JL . Finerenone, a non-steroidal mineralocorticoid receptor antagonist, reduces vascular injury and increases regulatory T-cells: studies in rodents with diabetic and neovascular retinopathy. Int J Mol Sci. (2023) 24:2334. doi: 10.3390/ijms24032334, PMID: 36768656 PMC9917037

[32] Hickey FB Martin F . Diabetic kidney disease and immune modulation. Curr Opin Pharmacol. (2013) 13:602–12. doi: 10.1016/j.coph.2013.05.002, PMID: 23721739

[33] Lassila M Jandeleit-Dahm K Seah KK Smith CM Calkin AC Allen TJ . Imatinib attenuates diabetic nephropathy in apolipoprotein E-knockout mice. J Am Soc Nephrol. (2005) 16:363–73. doi: 10.1681/ASN.2004050392, PMID: 15625075

