# Supplementary figures and images for: Integrated bioinformatics and machine learning reveal pan-apoptosis and immune infiltration signatures in diabetic nephropathy
Source: Front Immunol. 2025 Dec 17;16:1659065. doi: 10.3389/fimmu.2025.1659065 (PMC12753429; doi:10.3389/fimmu.2025.1659065)

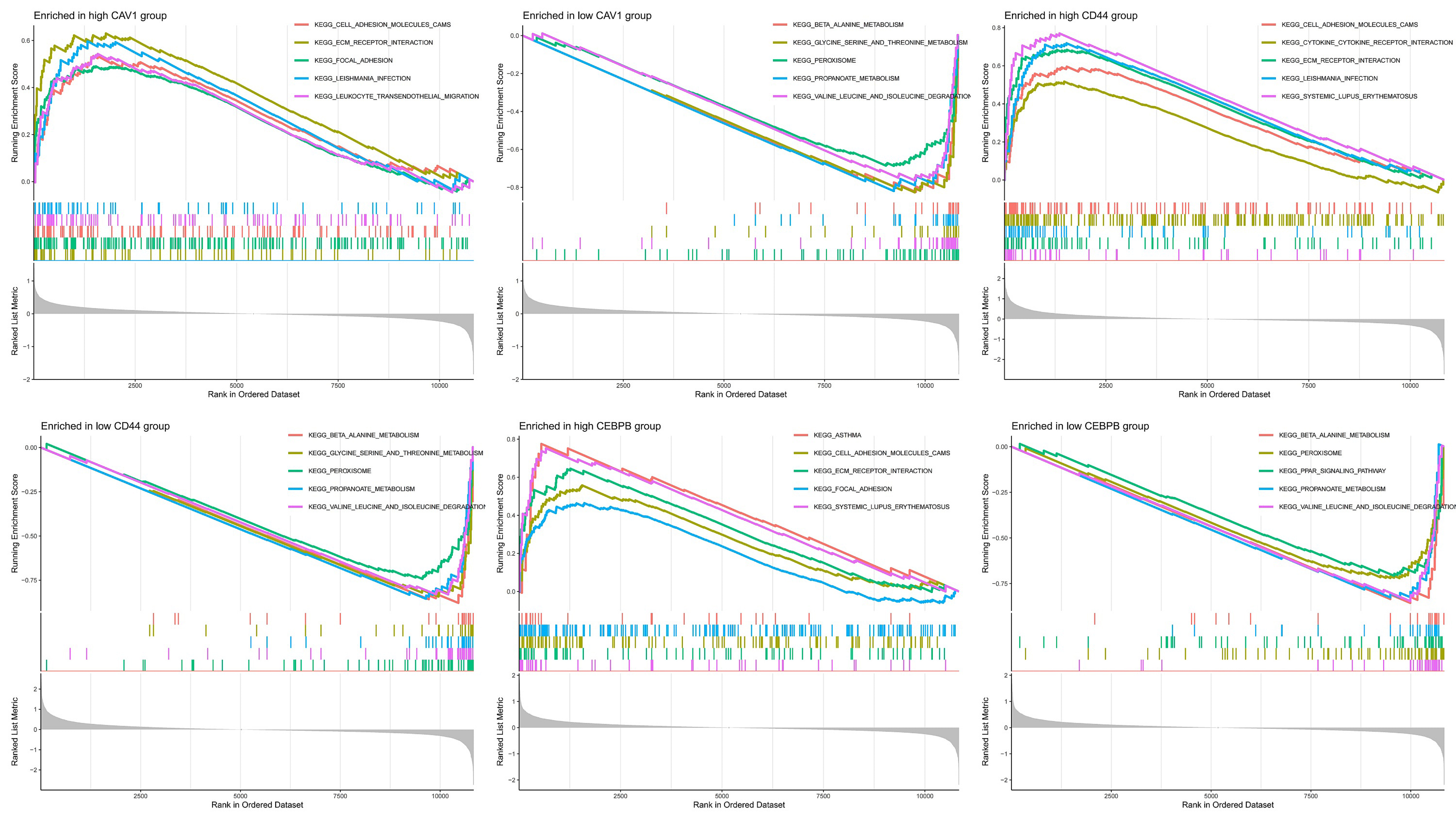

Supplement: Supplementary file 1 [file Image1.jpg]

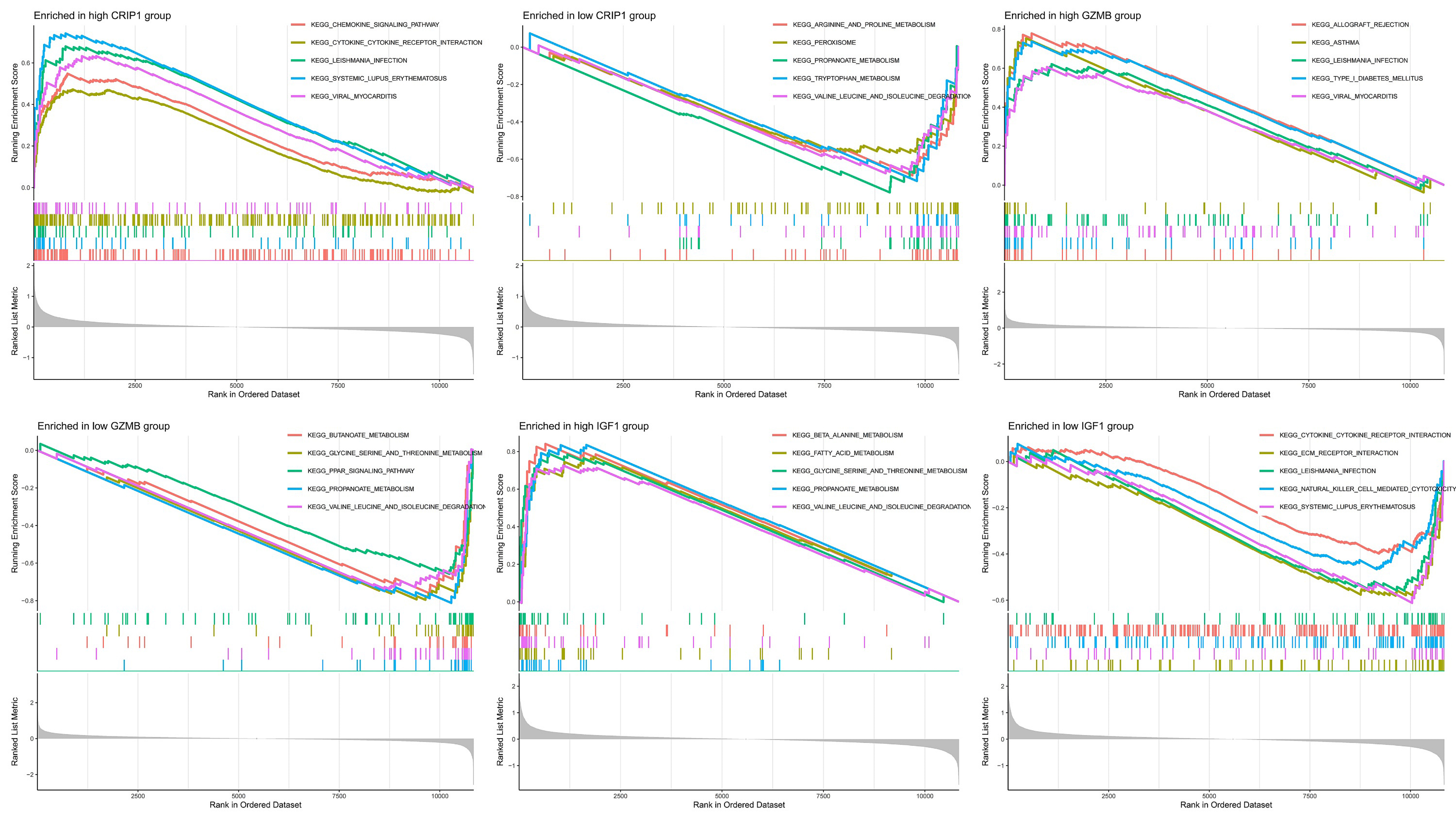

Supplement: Supplementary file 2 [file Image2.jpg]

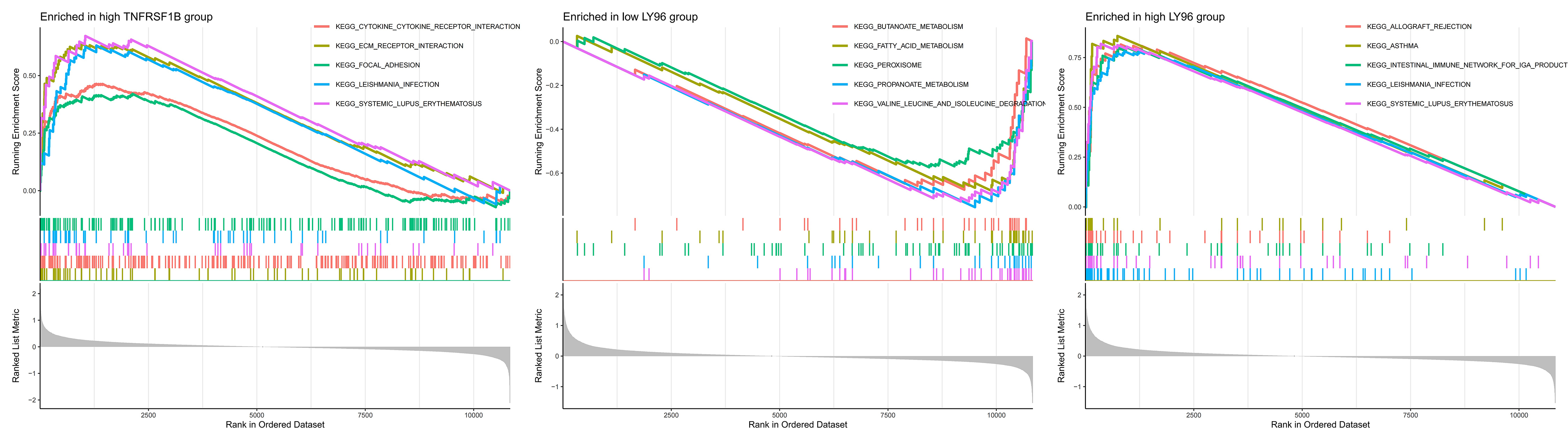

Supplement: Supplementary file 3 [file Image3.jpg]

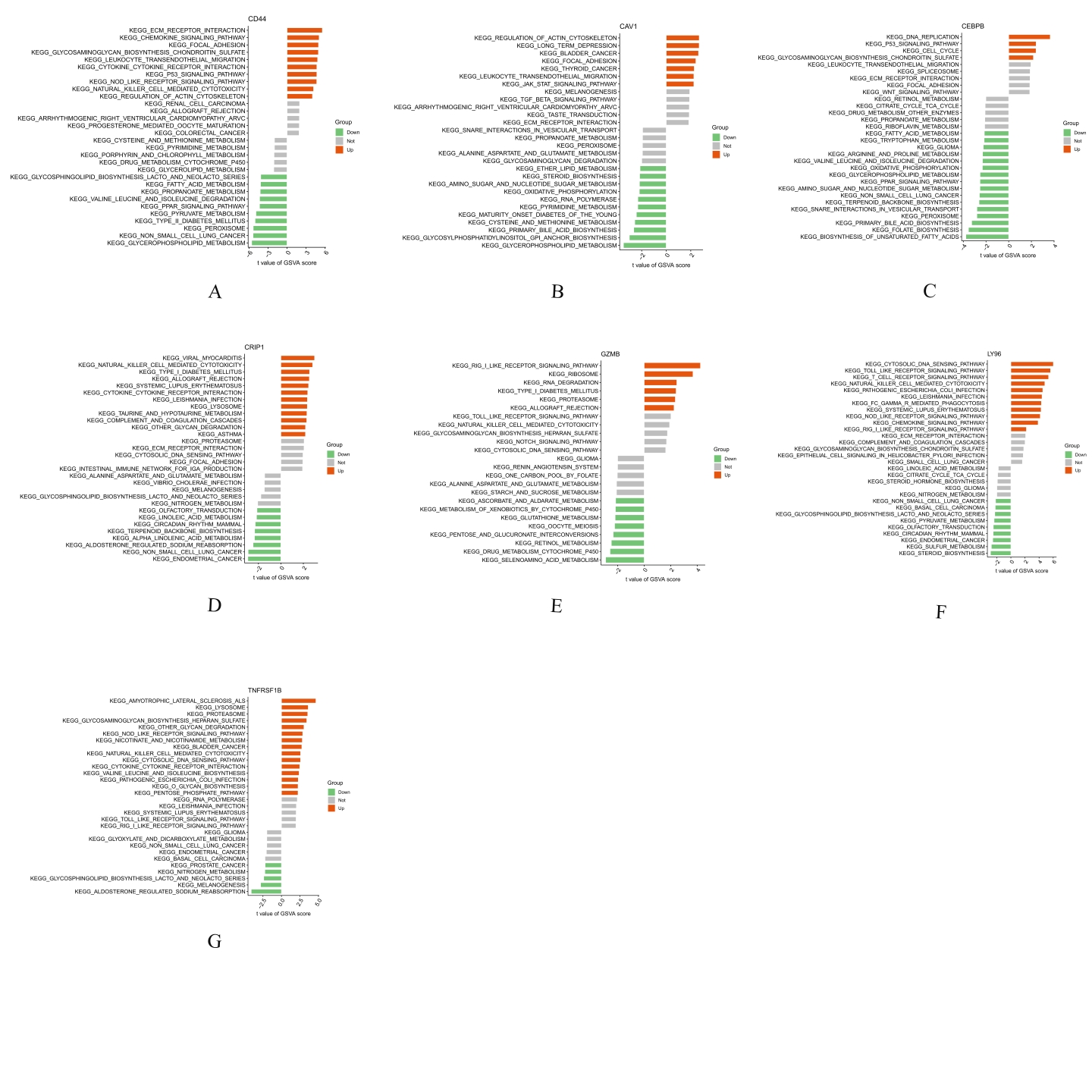

Supplement: Supplementary file 4 [file Image4.jpg]
